# Common Methylenetetrahydrofolate Reductase Polymorphism *MTHFR 677C>T* (rs1801133), Plasma Homocysteine, and Non-Valvular Atrial Fibrillation in Overweight/Obese Patients: Causality Indicated by Mediation and One-Sample Mendelian Randomization Analysis

**DOI:** 10.3390/diagnostics15222870

**Published:** 2025-11-12

**Authors:** Rea Levicki, Vladimir Trkulja, Vedran Pašara, Ivan Prepolec, Martina Matovinović, Lana Ganoci, Dragana Šegulja, Martina Lovrić Benčić, Tamara Božina

**Affiliations:** 1 Department of Cardiology, Požega General Hospital, 34000 Požega, Croatia; rlevicki@gmail.com; 2 Department of Pharmacology and Clinical Pharmacology, University of Zagreb School of Medicine, 10000 Zagreb, Croatia; vladimir.trkulja@mef.hr (V.T.); lana.ganoci@kbc-zagreb.hr (L.G.); 3 Department of Cardiovascular Diseases, University Hospital Centre Zagreb, University of Zagreb School of Medicine, 10000 Zagreb, Croatia; vedran.pasara@gmail.com (V.P.); miminamama@gmail.com (M.L.B.); 4 Department of Internal Medicine, University Hospital Centre Zagreb, 10000 Zagreb, Croatia; martina_10000@yahoo.com; 5 Faculty of Kinesiology, University of Zagreb, 10000 Zagreb, Croatia; 6 Department of Laboratory Diagnostics, University Hospital Centre Zagreb, 10000 Zagreb, Croatia; dsegulja@kbc-zagreb.hr; 7 Department of Medical Chemistry, Biochemistry and Clinical Chemistry, University of Zagreb School of Medicine, 10000 Zagreb, Croatia

**Keywords:** homocysteine, non-valvular atrial fibrillation, *MTHFR 677C>T*, *PITX2 C>T*, *KCNE1 112A>G*

## Abstract

**Background/Objectives**: The causal role of homocysteine (tHcy) in atrial fibrillation (AF) is unclear. To (re)explore the causal effect of tHcy in non-valvular AF (NVAF). **Methods**: In a case–control study in overweight/obese adults, cases were patients with NVAF and controls were their peers without AF. They were assessed for clinical, laboratory, and echocardiographic particulars and were genotyped for *MTHFR 677C>T* (rs1801133), *PITX2 C>T* (rs2200733), and *KCNE1 112A>G* (rs1805127) polymorphisms. We employed a conventional case–control, mediation analysis, and one-sample Mendelian randomization (MR) analyses to evaluate forward and reverse tHcy-NVAF associations. **Results**: We enrolled 180 cases and 179 controls. With an extensive confounder control (i) the *MTHFR 677C>T* variant allele associated with higher tHcy; (ii) *PITX2 C>T* variant allele associated with NVAF while *KCNE1 112A>G* did not; (iii) *MTHFR* variant associated with NVAF indirectly, through tHcy assuming wild type but not variant genotype (exposure–mediator interaction); (iv) considering all subjects, tHcy associated with NVAF through the effect on renal function and NT-proBNP levels (no exposure–mediator interaction); (v) considering *MTHFR* wild-type subjects (*n* = 160), tHcy “directly” strongly associated with NVAF, and considering variant carriers (*n* = 199), it indirectly associated with NVAF and directly tended to associate with a lower probability of NVAF; (vi) in MR analysis (*MTHFR* SNP instrument), tHcy associated with NVAF; and vii) mediation and MR analyses [*PITX2* SNP (exposure/instrument)—NVAF, (mediator/exposure)—tHcy outcome] excluded the reverse tHcy-NVAF association. **Conclusions**: Data strongly support the causal role of tHcy in NVAF in overweight/obese patients and suggest that the effect might be modified by the *MTHFR 677C>T* variant allele.

## 1. Introduction

Atrial fibrillation (AF) is the most common sustained cardiac arrhythmia in adults associated with considerable morbidity and mortality [1] and with increasing lifetime risk [2]. Numerous “triggers” or “perpetuators” [1] contribute to the occurrence of AF including demographics, health behaviors, inflammation, cardiovascular, and other health conditions [1,3,4]. Implementation of genome-wide association studies (GWAS), proteomics, metabolomics, transcriptomics, and Mendelian randomization (MR) analysis enabled the identification of some 140 genes associated with AF, many of which are likely causal by affecting cardiac electrophysiology, cardiomyocyte contractility/structure, and/or the development of the cardiac tissues [1,5,6,7,8]. Stratification systems combining (poly) genetic and conventional indicators have been developed to help identify people at high risk of AF [3,4,9], while understanding functional links between genetic markers and AF is hoped to result in the identification of novel drug targets [10]. Homocysteine (Hcy) is a non-essential amino acid derived from dietary methionine in the methylation cycle and is catabolized through the transsulfuration pathway or converted back to methionine by methionine synthase. The major pathway includes methyltetrahydrofolate as a methyl donor and vitamin B_12_ as a cofactor. Methyltetrahydrofolate is produced in the folate cycle by a reduction in methylenetetrahydrofolate by methylenetetrahydrofolate reductase (MTHFR) [11]. As recently reviewed [12], studies in human and animal cardiac tissues and animal models suggest Hcy as a causative factor in AF through effects on the atrial ion channels, cardiomyocyte remodeling, oxidative stress and related fibrosis, inflammation, and autonomic tone. Quality epidemiological studies have reported associations between high(er) total plasma Hcy (tHcy) and incident or prevalent AF [13,14,15,16]. Plasma tHcy is higher in men than in women and increases with age, smoking, nutritional folic acid/vitamin B_12_ deficiencies, or rare inborn disorders of vitamin metabolism [11]. Thirteen single nucleotide polymorphisms (SNPs) are GWAS-associated with high(er) tHcy, some encoding enzymes in the homocysteine/folate metabolism or transport proteins, and some for which protein products are not known [17]. The strongest appears to be the association of a common polymorphism *MHTFR 677C>T* (rs1801133), the only *MTHFR* polymorphisms GWAS-associated with plasma tHcy [17]. In vitro, variant allele carriers (CT/TT) have 30–60% reduced MTHFR activity [18]. However, none of the tHcy-associated SNPs appears to be GWAS-associated with AF; hence, a recent two-sample MR study indicated that increased tHcy was not causal to AF [19], implying that an otherwise effective [20] reduction in tHcy by folic acid supplementation would not affect the probability of AF. Still, a causal role of tHcy in AF might not be conclusively excluded: (i) tHcy may vary over time, regardless of the *MTHFR 677C>T* genotype. With varying exposures, the inherent analogy between MR studies and randomized trials is dubious—unless the analysis accounts for variable exposure over time [21]; (ii) interpretation of MR studies is not straightforward when the instrument affects enzyme activity—both the substrate and the product may be affected and may have opposing effects on the outcome [22]. In a prospective cohort study in Sweden, for example, tHcy was associated with a higher risk of incident AF, whereas (higher) plasma methionine was associated with a reduced risk of AF [23]. To (re)explore the possible causal role of tHcy in AF, we conducted a case–control study where cases were overweight/obese patients with prevalent non-valvular atrial fibrillation (NVAF), and controls were their peers in whom—at the time of study participation—AF was excluded. We reasoned that the restriction to overweight/obese patients—albeit reducing generalizability—would serve the purpose by reducing confounding and possible effect modification: obesity is a strong risk factor for AF [1,3,24], likely causal [25], acting through mechanisms [26] similar to those proposed for tHcy in AF [12], and modifies the association between AF and certain GWAS AF-associated genes [27]. We also evaluated associations between (NV)AF and two additional common SNPs, since they were likely important confounders: *PITX2 C>T* (rs2200733) and *KCNE1 112A>G* (rs1805127). The former is an SNP with the strongest GWAS association with AF [5,6,7,28], with similar findings in case–control studies [29]. It is located in the proximity of the *PITX2* gene which encodes three isoforms of the paired-like homeodomain transcription factor pitx2. The *PITX2 C>T* variant allele is associated with a reduced pitx2c expression (predominant in the developing heart) and structural and electrophysiological changes [30,31] that are likely causal to AF through a complex interplay with external stressors [32]. The *KCNE1* gene is one of the five genes encoding the regulatory β-subunit of the slowly activating delayed rectifying potassium current (IKs)—two rare mutations [G25V, G60D (gain-of-function)] are associated with the early-onset AF [33]. The *112A>G* SNP has not been GWAS-associated with AF [5,6,7,8]. Candidate-gene studies yielded heterogeneous results [34], but in some studies in European subjects, it was associated with prevalent NVAF [35], early-onset AF [36], or with incident AF after cardiac surgery [37].

## 2. Materials and Methods

### 2.1. Participants

The participants in this prevalent case–control study were recruited between 20 December 2021 and 16 March 2024 at the Department of Cardiology, Zagreb University Hospital Center, and Cardiology Unit, Department of Internal Medicine, County Hospital Požega, Croatia. This study was conducted in line with the ethical guidelines of the 1975 Declaration of Helsinki, and was approved by the Ethics Committees of the School of Medicine, University of Zagreb, (reg. number 380-59-10106-20-111/125; class 641-01/20-02/01, 29 September 2020) and the University Hospital Centre Zagreb (class 8.1-20/142-2; number 02/21 AG, 7 September 2020). All participants provided informed consent for study participation and for use of their anonymized data for research purposes/publishing.

All participants met common criteria: (i) age ≥18 years; (ii) non-related to other study participants; (iii) signed informed consent; (iv) body mass index ≥25 kg/m^2^ (office-measured weight and height with clothes and without shoes); and (v) free of coronary artery disease, chronic heart, kidney, and liver failure, obstructive sleep apnea, ongoing or recently treated malignancy, acute illness, advanced chronic obstructive pulmonary disease, chronic inflammatory conditions, moderate–severe mitral stenosis, congenital heart diseases, mechanical circulatory support, and heart transplantation. Patients with NVAF (European Society of Cardiology criteria) [1] were recruited among the outpatients managed at the participating sites. They underwent medical examination and a review of medical histories, transthoracal echocardiography, and provided blood samples for standard hematology/biochemistry panels, N-terminal pro B-type natriuretic peptide (NT-proBNP), plasma folate and tHcy concentrations, and for genotyping of the tested polymorphisms. Controls were subjects in whom (NV)AF was excluded based on medical histories, physical examination, 12-lead electrocardiogram repeated twice over a 15–30-day period, during which they provided blood samples, and who underwent transthoracal echocardiography. They were accrued among the staff at the participating institutions or as outpatients managed for hypertension. Absence of the coronary artery disease was confirmed based on medical histories/physical examination, risk factors, treadmill test, invasive coronarography or multislice computed tomography coronarography, as individually indicated. Other exclusionary comorbidities were assessed based on medical histories and clinical, laboratory, and echocardiographic evaluations. All patients were managed by two licensed cardiologists (RL, MLB).

### 2.2. Biochemistry

Standard clinical laboratory analyses were performed at the institutional laboratories. For quantification of homocysteine and folate concentrations, participants had to abstain from vitamin supplements/fruit juices for at least 7 days before blood sampling. Plasma for tHcy measurement was immediately stored at −20 °C until quantification at the department of clinical laboratory diagnostics, University Hospital Center Zagreb, using REF09P2820 reagent kit by the chemiluminescent microparticle immunoassay on fully automated analytical system Alinity ii (Abbott Laboratories, Chicago, IL, USA), as per manufacturer’s instructions. Immunoassay’s specification was verified using Multichem IA Plus (lot 032807240) (Technopath Clinical Diagnostics, Ballina, Ireland) multi-analyte control samples at three concentration levels.

### 2.3. Genotyping

Genomic DNA was extracted from 3 mL of whole blood taken in a K3EDTA tube (Vacuette, Greiner BioOne International AG, Kremsmünster, Austria) using the QIAamp DNA Blood Mini Kit (Qiagen, Hilden, Germany) according to the manufacturer’s protocol. Genotyping was performed using TaqMan^®^ SNP Genotyping Assays for *MTHFR C677T* (rs1801133; assay ID: C___1202883_20), *PITX2* (rs2200733; assay ID: C__16158671_10), and Custom TaqMan^®^ SNP Genotyping Assay for *KCNE1 G38S* (rs1805127), with TaqMan^®^ Universal PCR Master Mix (Applied Biosystems, Carlsbad, CA, USA) by real-time PCR genotyping on the 7500 Real-Time PCR System (Applied Biosystems, Carlsbad, CA, USA), according to the manufacturer’s instructions.

### 2.4. Data Analysis

We first estimated associations between the prevalent NVAF and *MTHFR 677C>T*, plasma tHcy, and *PITX2 C>T* and *KCNE1 112A>G* SNPs in conventional case–control type of analysis, where one exposure at the time was considered exposure of interest and the other three were covariates. We then employed mediation and one-sample Mendelian randomization/instrumental variable (MR/IV) analysis to detangle the relationship between *MTHFR* polymorphism, tHcy, and NVAF. For additional covariate adjustments, we considered plasma folate concentrations and known conventional risk factors for AF [1,3,24,38,39,40,41]: age, sex, current smoking, obesity (vs. overweight), pre-existing diagnosis of hypertension, actual blood pressure, diabetes mellitus, C-reactive protein (CRP), triglycerides, low-density lipoprotein cholesterol (LDL-C), NT-proBNP, creatinine, and urea (details in Supplementary Material A, A.1).

In the conventional case–control analysis (details in Supplementary Material A, A.2 and Figure S1) we (i) used covariate balancing by optimization-based weighting [42,43] to achieve balance between cases and controls on as many characteristics as possible (implemented in package *WeightIt* [44] in R version 4.5.0 [45]); (ii) variables that could not be adequately balanced were submitted for principal components analysis (PCA) for dimension reduction (combined categorical and continuous [46], package *PCAmixdata* [47] in R) and were included in multivariable logistic models fitted to weighted data with robust standard error estimation (SAS 9.4 for Windows, Cary, NC, USA). Generated estimates (odds ratios, ORs) were assessed for sensitivity to bias (we determined E-values [48] and estimates corrected for residual confounding and other biases [49], as implemented in packages *Evalue* [48] and *episensr* [50] in R). Estimates were also expressed as relative risks (RR) [51] for clearer relation to the E-values.

For the purpose of mediation and MR/IV analysis (details in Supplementary Material A, A.3), we first estimated the effect of the *MTHFR 677C>T* polymorphism on tHcy and repeated PCA with a larger number of covariates to reduce their dimensionality. Mediation analysis (causal [52], implemented in package *CMAverse* [53] in R, or traditional [54], implemented using macro Process in SAS 9.4 [54]) was used to assess forward association between *MTHFR* SNP or tHcy as exposures and NVAF and to assesses reverse association between tHcy and NVAF in a model using *PITX2 C>T* SNP as an exposure, NVAF as a mediator, and tHcy as the outcome. One-sample MR/IV analysis [55] (package *OneSampleMR* [56] in R) was used to assess forward association between tHcy (exposure) and NVAF using *MTHFR* SNP as an instrument and to assess reverse association between tHcy and NVAF in a model with NVAF as an exposure, tHcy as the outcome, and *PITX2 C>T* SNP as the instrument. E-values were provided for all estimates.

We planned to enroll equal numbers of cases and controls (around 185 each) sufficient to replicate (if they existed) the reported strengths of association between prevalent AF and *PITX2* SNP (OR ≥ 1.9 (or ≤0.53) in conventional prevalent case–control analysis (details in Supplementary Material A, A.4) [57]. We used package *genetics* (R) [58] to test the Hardy–Weinberg equilibrium (exact HWE test).

## 3. Results

### 3.1. Patient Characteristics

All subjects in the present study were Europeans of Slavic descent (Croatian nationals). Of the included 180 cases and 179 controls, the former were more commonly men (66.1% vs. 35.2%), with pre-existing hypertension (91.7% vs. 45.8%), were older (65 vs. 47 years), had higher blood pressure, NT-proBNP, creatinine, urea, and C-reactive protein (CRP) concentrations, and lower triglycerides and LDL-C (Table 1), whereas prevalence of obesity (44.8%), current smoking (17.6%), and diabetes (17.6%) was comparable in cases and controls (Table 1). The distribution of cases and controls across the *MTHFR 677C>T* genotypes was similar (Table 1). Plasma tHcy was higher (11.6 vs. 9.5 µmol/L, d = 0.665), and folate concentrations appeared moderately higher (15.9 vs. 13.9 nmol/L, d = 0.358) in cases than in controls (Table 1), but higher folate was associated with lower tHcy both in cases and in controls (Supplementary Material B, Figure S2). Cases were more commonly *PITX2 C>T* variant allele carriers than controls (43.9% vs. 29.0%) (Table 1), while distribution across the *KCNE1 112A>G* genotypes was similar (Table 1). Cases and controls variably differed in several other laboratory (Supplementary Material B, Table S1) and echocardiographic indices (Supplementary Material B, Table S2).

### 3.2. Conventional Case–Control Analysis

Weighing consistently enabled adequate balance between cases and controls regarding the SNPs (when covariates), folate concentrations, current smoking, BMI category, and diabetes (details in Supplementary Material C, C.1, Tables S3–S6). Of the covariates that could not be balanced, age, sex, tHcy (when a covariate), and CRP were treated as individual variables, while the remaining ones were subjected to PCA to identify three components (details in Supplementary Material C, C.2, Figure S3): “Blood pressure” (actual blood pressure, pre-existing hypertension), “Renal-BNP” (creatinine, urea, and NT-proBNP), and “Lipid” (LDL-C, triglycerides). Plasma tHcy partially correlated with male sex, older age, and higher (worse) “Renal-BNP” but not with other covariates (Supplementary Material C, C.3, Table S7).

In unadjusted analysis, higher tHcy and *PITX2 C>T* variant alleles were associated with NVAF, whereas variant alleles *MTHFR 677C>T* and *KCNE 112A>G* were not (Table 2). The relationships between the three SNPs and NVAF remained unchanged in partially (balanced data + adjustment for tHcy) and in fully adjusted analysis (balanced data + all covariates) (Table 2). tHcy was associated with NVAF in balanced data with additional adjustment for CRP, sex, “Blood pressure”, and “Lipid” (OR = 1.75, 95%CI 1.26–2.42) but not with further adjustment for age and “Renal-BNP” (fully adjusted) (Table 2). Fully adjusted ORs were somewhat altered after correction for hypothetical residual confounding and misclassification bias, but qualitative conclusions were not changed (Supplementary Material C, C.4, Table S8).

### 3.3. Effect of MTHFR 677C>T on Plasma tHcy

**Figure 1:**
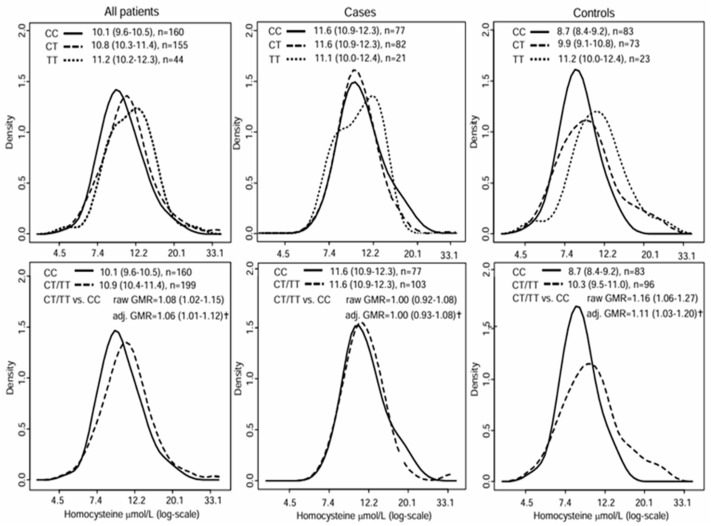
Effect of the *MTFHR 677C>T* polymorphism on plasma homocysteine concentrations. Panel columns refer to all patients (**left**), cases (**middle**), and controls (**right**). Panel rows refer to genotypes (upper, CC, CT, and TT) and to variant allele carriers and wild-type subjects (lower, CT/TT, or CC). Depicted are geometric mean homocysteine concentrations (95%CI) and numbers of patients (n) by genotype-based subset. Depicted are also differences between variant allele carriers and wild-type subjects (lower row) expressed as geometric means ratios (GMR) with 95%CI. ^†^ Adjusted for age, sex, current smoking, and folate concentrations.

Plasma tHcy increased across the *MHTFR* genotypes (from CC to TT), overall, and in controls but not in the cases (Figure 1). Variant allele carriers had a higher tHcy than wild-type subjects, overall (adjusted GMR = 1.06, 1.01–1.12), and in controls (GMR = 1.11, 1.03–1.20) but not in cases (GMR = 1.00, 0.93–1.08) (Figure 1). Cases differed from controls regarding tHcy in other aspects; as well, age, sex, folate, current smoking, and the *MTHFR* polymorphism explained only a minor part of tHcy variability in the former and a considerable part in the latter (Table 3); men had higher tHcy than women in controls but not in cases (Table 3); and the inverse association between folate and tHcy was 40% weaker in cases than in controls (Table 3).

### 3.4. Principal Components Analysis for the Purpose of Mediation and MR/IV Analysis

Polymorphisms *MTHFR*, *PITX2* and *KCNE1*, tHcy, age, sex, and folate concentrations were considered as individual variables, while others were submitted to PCA to identify five components: “Blood pressure”, “Lipid”, “Renal-BNP”, “Diabetes” (pre-existing diabetes, fasting glucose), and “BMI-CRP” (body mass index category, CRP, and current smoking) (details in Supplementary Material D, Figure S4). Plasma tHcy partially correlated with male sex, older age, and higher (worse) “Renal-BNP” but not with other covariates (Supplementary Material D, Table S9).

### 3.5. Mediation Analysis (Details in Supplementary Material E, Table S10 and Figure S5)

In the *MTHFR* (exposure)–tHcy (mediator)–NVAF (outcome) analysis (Figure S5A), the pure natural indirect effect (PNIE) indicated a higher risk of NVAF with higher tHcy under the wild-type *MTHFR* genotype, regardless of whether “Renal-BNP” was a confounder (Table 4, Model 1: RR = 1.074, 95%CI 1.025–1.116) or was excluded from the model (since it was likely a mediator) (Table 4, Model 2: RR = 1.100, 1.042–1.146). Pure total indirect effect (TNIE) (assumes *MTHFR* variant carriage) was around unity in both models (Table 4), reflecting the exposure–mediator interaction. There were no direct effects (PNDE, TNDE) of exposure (*MTHFR*) on the outcome (NVAF) in either model (Table 4). E-values for PNIE were relatively high. Correction for a hypothetical tHcy measurement error only slightly altered the estimates (Table 4).

In a traditional analysis with *MTHFR* exposure and two consecutive mediators (tHcy, “Renal-BNP”) (Figure S5B), exposure was indirectly associated with NVAF (Table 4, Model 3: OR = 1.032, 95%CI 1.016–1.093).

The tHcy (exposure)–“Renal-BNP” (mediator)–NVAF (outcome) analysis (Figure S5C) in all patients indicated a higher risk of NVAF, with higher tHcy mediated through its effect on “Renal-BNP” (Table 5, Model 1: PNIE RR = 1.233, 1.077–1.588; TNIE RR = 1.120, 1.006–1.646) and no direct effect of tHcy on NVAF (PNDE, TNDE) (Table 5, Model 1). The analysis in *MTHFR* wild-type subjects indicated a strong direct effect of tHcy on NVAF (Table 5, Model 2: PNDE RR = 3.929, 1.466–5.834; TNDE RR = 4683, 1.186–7.777) but no indirect effects (PNIE, TNIE) (Table 5, Model 2). Analysis in *MTHFR* variant carriers indicated a higher risk of NVAF, with higher tHcy mediated through “Renal-BNP” (Table 5, Model 3: PNIE RR = 1.189, 1.050–1.413; TNIE RR = 1.235, 1.085–1.755). Direct effects numerically indicated a lower risk of NVAF with higher tHcy (Table 5, Model 3: PNDE RR = 0.807, TNDE RR = 0.838) but with wide confidence intervals. All E-values for identified effects were high. Correction for a hypothetical tHcy measurement error only slightly altered the estimates (Table 5).

In the assessment of the reverse tHcy-NVAF association (*PITX2* exposure–NVAF mediator–ln[tHcy] outcome) (Figure S5D), there was no effect of exposure on the outcome regardless of whether “Renal-BNP” was a confounder or not in the model (since it was a potential collider) (Table 6).

### 3.6. One-Sample MR/IV Analysis

Selection of covariates (potential confounders) in the MR/IV models is elaborated in Supplementary Material F (Tables S11 and S12).

In the model for forward tHcy-NVAF association, tHcy was associated with a higher risk of NVAF (Table 7, Model 1: RR = 2.333, 1.063–5.120) with the expected effects of covariates (higher risk in *PITX2* variant carriers vs. wild type and in men vs. women; lower risk with higher “Lipid”).

In the model for reverse tHcy-NVAF association, NVAF had no effect on tHcy (Table 7, Model 2: RR = 1.045, 0.573–1.907).

## 4. Discussion

The present study strongly supports the causal role of tHcy in NVAF in overweight/obese people: complementary analytical approaches consistently point in this direction. Data also illustrate how mediation analysis may help disclose the link between the *MTHFR 677C>T* SNP and the outcome, which typically remained obscured in the conventional cohort or case–control studies [13,16,23] and indicate that the *MTHFR 677C>T* genotype might modify the effect of tHcy.

### 4.1. Effect of tHcy on NVAF

In the conventional analysis, tHcy was strongly associated with NVAF after controlling for a number of the risk factors for (NV)AF [1,3,24,30,31,32,38,39,40,41] (by exclusion criteria and analytically), but with further adjustment for age and “Renal-BNP”, the association was lost (Table 2). Attenuation of the tHcy–outcome association after controlling for age (affects tHcy [11] and the risk of AF [1]) was expected [13,14]. On the other hand, we considered that “Renal-BNP” should be viewed as a mediator, since both renal function and NT-proBNP are likely causally affected by tHcy [59,60,61]. In a (fully adjusted) mediation analysis, tHcy (exposure) associated with NVAF via “Renal-BNP” (mediator) (Table 5, Model 1), as indicated by the natural indirect effects (PNIE, TNIE). The total effect (product of the indirect and direct components [62]) appeared strong (point RR = 1.406) but imprecisely estimated (lower CI limit at 0.832). One-sample MR/IV analysis (*MTHFR 677C>T* instrument, tHcy exposure, and NVAF outcome), adjusted for potential confounders, also indicated tHcy-NVAF association (Table 7, Model 1). Importantly, adjusted mediation and MR analyses [*PITX2 C>T* (exposure or instrument, respectively)–NVAF (mediator or exposure, respectively)–tHcy (outcome)] excluded a reverse NVAF-tHcy association (Table 6, Table 7: Model 2).

### 4.2. MTHFR 677C>T Variant Allele Associates with NVAF

As in similar studies [13,16,23], in the conventional analysis, *MTHFR 677C>T* associated with higher tHcy but not with NVAF, although tHcy associated with NVAF. This appears in line with the GWAS association of this SNP with tHcy [17] but not with AF [19]. However, these observations are in contradiction regarding the causal role of tHcy: the former could be viewed as evidence of the causal tHcy effect, potentially indicating that tHcy concentration does not critically depend on *MTHFR 677C>T*, whereas the latter resulted in a two-sample MR analysis claiming no causal effect of tHcy on (NV)AF [19]. Considering the potential of the two-sample MR methodology to avoid confounding [55], one could assume that “conventional” epidemiological data [13,14,15,16,23] must have been biased, i.e., that there is no causal effect of tHcy on (NV)AF. Such a conclusion would be in contrast with the present one-sample MR/IV analysis. In this context, two points need to be addressed: the potential weak instrument bias and possible attenuation of exposure-outcome association with multiple mediators. In the case of weak instruments, two-sample MR studies are biased towards the null, whereas one-sample studies are biased away from the null [63]. However, this does not seem to be a plausible explanation of the pre-sent situation: (i) Although the effect of the *MTHFR 677C>T* variant allele on tHcy in adults is not especially marked ([13,16,23], current data]), it is a valid instrument—two-sample MR analyses have used it to demonstrate the causal effect of tHcy on small vessel ischemic stroke [64,65]; (ii) in one-sample MR/IV analysis, this bias can be minimized by accounting for possible confounders and by avoiding adjustments for variables on the exposure–outcome pathways [63], as we did in the present analysis. Hence, it is highly unlikely that the present estimate was relevantly inflated. Next, when the link between an exposure and an outcome includes multiple/additional mediators, the strength of their association could attenuate both in the MR and “conventional” analysis [66,67]. The present analysis suggests that when mediation is accounted for, the link between *MTHFR* and NVAF becomes apparent: in the traditional mediation analysis, the mediated effect of SNP—tHcy—“Renal-BNP”—NVAF appeared weak but obvious (Table 4, Model 3). The link was also disclosed in causal mediation analysis with *MTHFR 677C>T* as an exposure and tHcy as the mediator, as indicated by PNIE (Table 4, Model 1, and Model 2). This analysis introduced another complex point: modification of the effect of the mediator (tHcy) by exposure (*MTHFR*) illustrated by a difference between the two natural indirect effects—PNIE, which indicated an SNP-NVAF association via tHCy, and TNIE, indicating no association (Table 4).

### 4.3. MTHFR 677C>T Modifies the Effect of tHcy on NVAF

In a conventional multivariable analysis of two case–control studies in Sweden [68], higher tHcy consistently associated with prevalent coronary artery disease (CAD). How-ever, the association appeared strong in *MTHFR* wild-type subjects and was negligible/had no association in variant allele carriers. While plasma tHcy is markedly affected by a number of “conventional factors” [11], the authors hypothesized [68] that, in people with a long-term genetically mild–moderately increased tHcy (e.g., *MTHFR 677C>T* variant carriers), the impact of these “other factors” eventually attenuates. Hence, they suggested that, in the wild-type subjects (where the “genetic basis” favors lower basic tHcy), a reduction in plasma tHcy (e.g., by folic acid) might be likely to reduce the risk of CAD, whereas in variant carriers (with “genetic basis” favoring long-term, higher basic tHcy) this was unlikely. A meta-regression analysis of randomized trials (RCTs) of folic acid for a reduction in the risk of cardio-/cerebrovascular disease (CVD) [69] indicated that the intervention reduced the risk of CVD in people with lower baseline tHcy (e.g., ≤10 µmol/L) (among whom the prevalence of *MTHFR 677C>T* wild-type subjects might be reasonably assumed), whereas in those with higher baseline tHcy (prevalence of variant carriers is reasonable to assume) there was no benefit or even harm was suggested [69]. Results of other meta-analyses of RCTs of folic acid for the prevention of stroke [70] or CVD [71,72] essentially agree with these observations: risk reduction was seen in patients with no previous strokes/CVD events but not in those with a history of such events. A study in Sweden [23] indicated that higher baseline tHcy independently contributed to a greater rate of CVD multimorbidity accumulation over time. Hence, patients without a history of stroke/CVD who benefited from the treatment could be reasonably viewed as those with a likely lower baseline tHcy and prevalence of *MTHFR 677C>T* wild-type subjects, whereas those with a history of stroke/CVD who did not benefit could be reasonably viewed as those with a likely higher baseline tHcy and prevalence of variant allele carriers. In the present analysis, modification of the tHcy effect by the SNP is illustrated by several findings. First, it is the difference between PNIE and TNIE in mediation analysis, with SNP as the exposure and tHcy as the mediator (Table 4). In causal mediation [52], the natural indirect effect (NIE) implies that “switching” the exposure from a control to an active level “switches” the mediator from a control to the active level, which then affects the outcome, and is estimated while the “direct” effect of exposure is blocked by being fixed in all subjects either at the control level (e.g., as if all were *MTHFR* wild type) (PNIE) or at the active level (e.g., as if all were variant carriers) (TNIE). Hence, PNIE estimates the effect of an increase in tHcy assuming that all subjects are *MTHFR* wild type and hence have basic tHcy corresponding to the control (wild-type) level of *MTHFR* SNP (i.e., “lower” basic tHcy), whereas TNIE estimates it assuming that all are *MTHFR* variant carriers and have basic tHcy corresponding to the active (variant carrier) level of the *MTHFR* SNP (i.e., “higher” basic tHcy). A further inherent facet of this effect modification (the other side of the same coin) are the present observations about differences between controls and cases regarding the effects of several “conventional” factors on tHcy (Table 3): in the former, they explained a large portion of tHcy variability and strongly associated with tHcy concentrations; in the latter, they explained only a minor portion of tHcy variability and weakly or not at all associated with tHcy concentrations. This is in agreement with the hypothesis generated in the Swedish case–control studies in patients with CAD [68]. In contradiction, one previous prevalent case–control study [16] suggested that the tHcy levels increased with the *MTHFR 677C>T* variant allele in both patients with NVAF and in controls. However, in that study, cases and controls were by design matched on age and sex, which were not included in the analytical models [16]. Such procedures do not resolve confounding and introduce selection bias (impose identical distribution of the exposure of interest to cases and controls in the source population) [73,74]. The third indicator of modification of the tHcy effect on NVAF by the *MTHFR 677C>T* polymorphism is the differential effect of tHcy (exposure) in mediation analysis (“Renal-BNP” is the mediator) separately in *MTHFR 677C>T* wild-type subjects and in variant carriers. In the former (Table 5, Model 2), analysis indicated strong direct effects (PNDE, TNDE) of tHcy on NVAF, thus indicating *MTHFR 677C>T* wild-type subjects (who also had lower tHcy—Figure 1) as those in whom a tHcy reduction might confer the benefit of NVAF risk reduction. In contrast, the same type of analysis in the latter (Table 5, Model 3) indicated a null total effect, and PNIE and TNIE indicated a moderate mediated (via “Renal-BNP”) association between higher tHcy and NVAF, whereas direct effects (PNDE, TNDE) indicated a possible milder association between higher tHcy and a lower probability of NVAF.

### 4.4. Hypothesis: In MTHFR 677C>T Variant Carriers, tHcy Might Increase and Decrease the Risk of NVAF

The null total effect of tHcy on NVAF in the mediation analysis in *MTHFR 677C>T* variant carriers (Table 5, Model 3) has two components: indirect effects (PNIE, TNIE), both indicating association between (higher) tHcy and NVAF (RR = 1.189, 1.050–1.413; RR = 1.235, 1.085–1.755, respectively), and direct effects (PNDE, TNDE), both indicating a tendency of reduced probability of NVAF with higher tHcy (point RR = 0.807 and RR = 0.838, respectively) but estimated with less precision (CIs extending to >1.0). It is tempting to speculate that there could be two paths from tHcy to NVAF, one via “Renal-BNP” to increase the risk of NVAF and another one acting in the opposite direction. In a Swedish cohort study [23], higher plasma tHcy was associated with a greater rate of accumulation of incident AF over time while higher plasma methionine tended towards a lower rate, but a higher methionine/tHcy ratio was associated with a reduced rate. It appears reasonable to hypothesize that the increased tHcy might have contributed not only to the higher incidence rate but also to the reduced one by, e.g., affecting the methionine levels. This hypothesis implies that there is modification of the tHcy effect and is in line with the elaborated evidence from randomized trials [69] that a tHcy reduction in people with higher baseline tHcy (who are likely predominantly *MTHFR 677C>T* variant carriers) might be harmful regarding the CVD risk. The hypothesis is elaborated in detail in Supplementary Material G (Figures S6–S8).

### 4.5. Other Findings

In line with expectations [5,6,7,28,29], *PITX2 C>T* strongly associated with NVAF. We used it to reduce confounding and as an instrument (MR) or as an exposure (mediation) to exclude reverse NVAF-tHcy association. We accounted for *KCNE1 112A>G* with the same intentions, but no association with NVAF was observed. This disagreed with the expectations but was not particularly surprising. Most of the previous studies were conducted in Chinese patients with highly heterogeneous results [34], and only one prevalent case–control study in Europeans addressed this SNP in relation to NVAF specifically [35].

### 4.6. Limitations

Generalizability of the present observations is limited, partly due to the properties of the source population (Europeans of Slavic descent residing in the catchment areas of the two participating institutions), partly due to the prevalent case–control design and a limited sample size, and in part was created on purpose (by inclusion/exclusion criteria) for the practical reasons of reducing confounding and effect modification. Still, characteristics of the cases and controls, and differences between them regarding classical NVAF risk factors (e.g., age, sex, hypertension, CRP, indicators of the renal function, NT-proBNP, LDL-C, and triglycerides) were generally in line with the expectations [1]. Next, the selection of study participants was unlikely to introduce bias (e.g., selection, collider [73,74]) since cases were consecutive NVAF outpatients, whereas the controls came from the same source population, were generally healthy (medical and non-medical staff from the participating institutions), or were outpatients with hypertension managed by the same investigators. The reports about the GWAS association of *MTHFR 677C>T* with hypertension have been contradictory, and conventional and MR studies support the conclusion of no causal effect of tHcy on hypertension [75,76,77]. Hence, the exposure of primary interest (tHcy) most likely did not affect the condition that brought the controls in contact with the investigators. Additionally, we consider it unlikely that the present estimates are affected by the Neyman’s (“incidence-prevalence”) bias, since it implies that the causal factor to disease occurrence (tHcy to NVAF) is also causal to disease-specific case fatality, which, in the present setting, is highly questionable. Even if so, one would expect that (higher) tHcy resulted in selective mortality, which would mean that the true tHcy-NVAF association (effect of tHcy) was stronger than reported here. Finally, by design and analytically, we accounted for a number of potential confounders. Reasonably high E-values indicated that those unaccounted for (e.g., other genetic factors, physical activity, and other vitamin B concentrations and supplementation) would have needed to have a rather strong cumulative effect to explain away the present estimates. Taken together with the fact that estimates corrected for strong hypothetical residual confounding and misclassification bias (outcome) or a hypothetical error in the measurement of tHcy that did not materially change the qualitative conclusion, this supports a fair level of internal validity of the generated estimates.

## 5. Conclusions

Present observations in a sample of overweight or obese adults support a causal role of plasma tHcy in NVAF and suggest that the effect is modified by the *MTHFR 677C>T* variant allele. Data justify further methodologically multifaceted research to detangle, confirm, or reject a casual effect of tHcy on AF across a variety of patient (ethnicity, demographics, and comorbidities) and disease characteristics (e.g., pattern and duration, recurrence after therapeutic interventions).

## Figures and Tables

**Table 1:** Characteristics of patients with (cases) and without (controls) non-valvular atrial fibrillation. Data are count (%), median (range, quartiles for cases/controls), geometric mean (%coefficient of variation) for ln-transformed variables, and standardized mean differences (d) between cases and controls.

	All	Cases	Controls	d
N	359	180	179	---
Age (years)	59 (21–87)	65 (59–79)	47 (39–59)	1.393
Men	182 (50.7)	119 (66.1)	63 (35.2)	0.650
Hypertension	247 (68.8)	165 (91.7)	82 (45.8)	1.138
3 × sitting office systolic BP (mmHg)	130 (120–150)	145 (130–160)	130 (120–130)	1.232
3 × sitting office diastolic BP (mmHg)	80 (75–90)	90 (80–95)	80 (70–80)	1.058
Ln[N-terminal proBNP (ng/L)]	117.7 (185)	249.3 (185)	55.3 (65.1)	1.569
Ln[Creatinine (μmol/L)]	79.7 (23.8)	87.2 (23.3)	72.8 (20.6)	0.825
Ln[Urea (mmol/L)]	5.53 (29.2)	6.20 (30.2)	4.92 (22.6)	0.887
Ln[C-reactive protein (mg/L)]	1.9 (89.5)	2.2 (108)	1.7 (66.8)	0.354
LDL-C (mmol/L)	3.4 ± 1.0	3.2 ± 1.1	3.6 ± 0.8	−0.416
Ln[Triglycerides (mmol/L)]	1.44 (50.7)	1.37 (49.1)	1.52 (51.8)	−0.226
Diabetes mellitus	63 (17.6)	38 (21.1)	25 (14.0)	0.189
Body mass index (kg/m^2^)	29.4 (25.0–57.4)	29.7 (27.5–32.0)	29.4 (26.6–32.7)	−0.134
Overweight	198 (55.2)	96 (53.3)	102 (57.0)	−0.073
Obese	161 (44.8)	84 (46.7)	77 (43.0)	0.073
Current smokers	63 (17.6)	35 (19.4)	28 (15.6)	0.100
Fully abstain from alcohol	315 (87.7)	139 (77.2)	176 (98.3)	−0.680
Coronary disease excluded by the following:				
History, examination and no risk factors	78 (21.7)	0	78 (43.6)	---
Treadmill stress test	222 (61.8)	128 (71.1)	94 (52.5)	---
MSCT angiography	24 (6.7)	19 (10.6)	5 (2.8)	---
Coronarography	35 (9.7)	33 (18.3)	2 (1.1)	---
*Polymorphisms and metabolites of interest*				
*MTHFR 677C>T* (rs1801133) ^1^				
CC	160 (44.6)	77 (42.7)	83 (46.4)	−0.072
CT	155 (43.2)	82 (45.5)	73 (40.8)	0.097
TT	44 (12.2)	21 (11.7)	23 (12.8)	−0.036
Variant carriers	199 (55.4)	103 (57.2)	96 (53.6)	0.072
Variant allele frequency	0.338	0.289	0.332	---
Ln[Homocysteine (μmol/L)]	10.5 (31.1)	11.6 (27.1)	9.5 (31.8)	0.665
Ln[Folate(nmol/L)]	14.8 (39.5)	15.9 (34.8)	13.9 (42.7)	0.358
*KCNE1 c.112 A>G* (rs1805127) ^1^				
AA	79 (22.0)	44 (24.4)	35 (19.6)	0.118
AG	171 (47.6)	78 (43.3)	93 (52.0)	−0.173
GG	109 (30.4)	58 (32.2)	51 (28.5)	0.081
Variant carriers	280 (78.0)	136 (75.6)	144 (80.4)	−0.118
Variant allele frequency	0.458	0.461	0.454	---
*PITX2 C>T* (2200733) ^1^				
CC	228 (63.5)	101 (56.1)	127 (71.0)	−0.321
CT	115 (32.0)	69 (38.3)	46 (25.7)	0.273
TT	16 (4.5)	10 (5.6)	6 (3.3)	0.107
Variant carriers	131 (36.5)	79 (43.9)	52 (29.0)	0.312
Variant allele frequency	0.205	0.247	0.161	---

^1^ In Hardy–Weinberg equilibrium. BP—blood pressure; LDL-C—low-density lipoprotein cholesterol; MSCT—multislice computed tomography; and BNP—brain natriuretic peptide.

**Table 2:** Association between *MTHFR 677 C>T* (rs1801133), plasma homocysteine (tHcy), *PITX2 C>T* (rs2200733), *KCNE1 C.112A>G* (rs1805127), and non-valvular atrial fibrillation (NVAF) [odds ratios (OR) and prevalence ratios (RR) for a comparison to E-values]: unadjusted, partially adjusted, and fully adjusted estimates. Shown are E-values for the fully adjusted estimates indicating association between the case status and the exposures.

	Unadjusted		Partially Adjusted ^1^		Fully Adjusted ^2^		
	OR (95%CI)	RR (95%CI)	OR (95%CI)	RR (95%CI)	OR (95%CI)	RR (95%CI)	E-value ^3^
*MTHFR 677 C>T* variant	1.16 (0.76–1.75)	1.08 (0.87–1.32)	1.01 (0.65–1.56)	1.00 (0.81–1.25)	0.98 (0.49–1.97)	0.99 (0.70–1.40)	---
tHcy (by 33% higher)	1.98 (1.52–2.59)	1.41 (1.23–1.61)	1.75 (1.26–2.42)	1.32 (1.12–1.56)	1.00 (0.69–1.45)	1.00 (0.83–1.20)	---
*PITX2 C>T* variant	1.91 (1.23–2.96)	1.38 (1.11–1.72)	2.11 (1.31–3.38)	1.45 (1.14–1.84)	2.39 (1.13–5.07)	1.55 (1.06–2.25)	2.31
*KCNE1 C.112A>G* variant	0.75 (0.45–1.24)	0.87 (0.67–1.11)	0.64 (0.37–1.09)	0.80 (0.61–1.04)	0.77 (0.37–1.59)	0.88 (0.61–1.26)	---

^1^ Analysis in weighted (balanced) data sets with additional adjustment for some of the individual covariates: tHcy in the case of polymorphisms; C-reactive protein (CRP), sex, “Blood pressure”, and “Lipids” from PCA in the case of tHcy. ^2^ Analysis in weighted (balanced) data sets with additional adjustment for all individual covariates. ^3^ E-value indicates strength of association on a risk ratio scale that a confounding set needs to have both with the exposure and the outcome to explain away the observed exposure–outcome association. For example, E-value 2 (or 0.5) indicates that it would need to be twice more (or 50% less) prevalent among the exposed than among control subjects and would need to increase the probability of the outcome 2-fold (or reduce it by 50%). E-values are reported only for variables that appear to be associated with the prevalent NVAF in fully adjusted analysis. They refer to confounding effects needed to reduce a fully adjusted OR that was higher than 1.0 to 1.20 (OR = 1.20 corresponds to Cohen’s d = 0.1, i.e., “irrelevant effect”).

**Table 3:** The effects and explanatory contributions of factors known to affect plasma homocysteine concentrations differ in cases and controls. We conducted hierarchical linear regression analysis of ln(homocysteine) concentrations to illustrate the effects of demographics, smoking, folate concentrations and *MTHFR 677C>T* variant allele separately in cases and in controls to depict differences in the size of their effects and contribution to variability of homocysteine concentrations in the two patient subsets. Shown are adjusted R^2^ values, change in R^2^ values for the models (formed by gradual introduction of blocks of explanatory variables), and geometric means ratios (GMR) with 95%CI as measures of effects.

	Cases			Controls		
	Adj. R^2^	ΔR^2^	GMR (95%CI)	Adj. R^2^	ΔR^2^	GMR (95%CI)
*Model 1*	0.063	0.063		0.193	0.193	
Age (5 years)			1.04 (1.02–1.06)			1.04 (1.02–1.06)
Male sex			1.06 (0.98–1.15)			1.21 (1.11–1.32)
*Model 2*	0.066	0.003		0.195	0.002	
Age (5 years)			1.04 (1.02–1.06)			1.05 (1.04–1.06)
Male sex			1.05 (0.97–1.14)			1.21 (1.11–1.32)
Current smoker			1.06 (0.96–1.17)			1.07 (0.96–1.20)
*Model 3*	0.109	0.043		0.333	0.138	
Age (5 years)			1.04 (1.02–1.06)			1.05 (1.04–1.06)
Male sex			1.04 (0.96–1.13)			1.20 (1.11–1.30)
Current smoker			1.06 (0.96–1.16)			1.05 (0.94–1.16)
Ln(folate)			0.84 (0.76–0.94)			0.75 (0.69–0.82)
*Model 4*	0.104	−0.005		0.358	0.025	
Age (5 years)			1.04 (1.02–1.06)			1.05 (1.03–1.06)
Male sex			1.04 (0.96–1.13)			1.19 (1.10–1.28)
Current smoker			1.06 (0.96–1.16)			1.05 (0.95–1.17)
Ln(folate)			0.84 (0.76–0.94)			0.76 (0.69–0.83)
*MTHFR* variant			1.00 (0.93–1.08)			1.11 (1.03–1.20)

**Table 4:** Summary of the adjusted † mediation analysis to estimate forward association between the *MTHFR 677C>T* polymorphism (*MTHFR*) as an exposure (variant allele carriage) and non-valvular atrial fibrillation (NVAF) as the outcome. Shown are results of the causal mediation analysis with plasma homocysteine (tHcy) as a mediator and with “Renal-BNP” (from the principal components analysis) considered a confounder (Model 1) or excluded from the model, since it was most likely a mediator (Model 2). Shown are also results from a traditional mediation model (Model 3) with two consecutive mediators—tHcy and “Renal-BNP”. Corrected refers to relative risks estimated in the causal mediation analysis with a correction for a hypothetical non-differential error in measurement of tHcy.

	RR/OR (95%CI)	E-Value	Corrected
Model 1 ‡ (causal): *MTHFR*—tHcy—NVAF (Renal-BNP included as a confounder)			
Pure natural direct effect (PNDE)	1.008 (0.805–1.255)	---	1.021
Total natural direct effect (TNDE)	0.927 (0.798–1.156)	---	0.933
Pure natural indirect effect (PNIE)	1.074 (1.025–1.116)	1.335	1.080
Total natural indirect effect (TNIE)	0.988 (0.968–1.000)	---	0.987
Total effect	0.995 (0.802–1.254)	---	1.008
Model 2 ‡ (causal): *MTHFR*—tHcy—NVAF (Renal-BNP not in the model)			
Pure natural direct effect (PNDE)	1.063 (0.850–1.243)	---	1.042
Total natural direct effect (TNDE)	0.965 (0.829–1.154)	---	0.945
Pure natural indirect effect (PNIE)	1.100 (1.042–1.146)	1.431	1.102
Total natural indirect effect (TNIE)	0.998 (0.972–1.019)	---	0.999
Total effect	1.062 (0.845–1.264)	---	1.041
Model 3 ‡ (traditional): *MTHFR*—tHcy-RenalBNP-NVAF			
Direct effect	1.127 (0.538–2.359)	---	---
Indirect effect	1.032 (1.016–1.093)	1.140	---
Total effect	1.129 (0.888–1.477)	---	---

† Variables included in all models as confounders of the exposure–mediator, mediator–outcome, and exposure–outcome effects: age, sex, *KCNE1* and *PITX2* polymorphisms, folate concentrations, and components identified in the principal components analysis: “Blood pressure”, “Diabetes”, “BMI-CRP”, and “Lipid”. ‡ In the causal mediation model, there was a significant exposure (*MTHFR*)*mediator (tHcy) interaction (*p* = 0.004 if Renal-BNP was included as a confounder and *p* = 0.003 if not in the model.) In the traditional model, there was a significant MTHFR*tHcy interaction (*p* = 0.002), but there was no interaction between the exposure and Renal-BNP as the mediator (*p* = 0.409).

**Table 5:** Summary of the adjusted † mediation analysis to estimate forward association between plasma homocysteine (tHcy) as an exposure and non-valvular atrial fibrillation (NVAF) as the outcome, with Renal-BNP from the principal components analysis as the mediator. Shown are results of the causal mediation analysis conducted in all patients (Model 1) and separately in *MTHFR 677C>T* wild-type subjects (Model 2) and in variant allele carriers (Model 3). Corrected refers to relative risks estimated with a correction for a hypothetical non-differential error in measurement of tHcy.

	RR (95%CI)	E-Value	Corrected
Model 1 ‡: All patients (*N* = 359)			
Pure natural direct effect (PNDE)	1.174 (0.639–1.578)	---	1.185
Total natural direct effect (TNDE)	1.171 (0.687–1.579)	---	1.151
Pure natural indirect effect (PNIE)	1.233 (1.077–1.588)	1.770	1.245
Total natural indirect effect (TNIE)	1.120 (1.006–1.646)	1.690	1.210
Total effect	1.406 (0.832–2.019)	---	1.434
Model 2 ‡: *MTHFR 677C>T* wild-type subjects (*n* = 160)			
Pure natural direct effect (PNDE)	3.929 (1.466–5.834)	7.322	4.616
Total natural direct effect (TNDE)	4.683 (1.186–7.777)	8.838	5.472
Pure natural indirect effect (PNIE)	1.215 (0.843–1.441)	---	1.246
Total natural indirect effect (TNIE)	1.448 (0.663–2.062)	---	1.477
Total effect	5.691 (1.503–10.20)	10.86	6.817
Model 3 ‡: *MTHFR 677C>T* variant carriers (*n* = 199)			
Pure natural direct effect (PNDE)	0.807 (0.673–1.111)	---	0.789
Total natural direct effect (TNDE)	0.838 (0.718–1.310)	---	0.823
Pure natural indirect effect (PNIE)	1.189 (1.050–1.413)	1.664	1.208
Total natural indirect effect (TNIE)	1.235 (1.085–1.755)	1.773	1.261
Total effect	0.997 (0.754–1.434)	---	0.995

† Variables included in all models as confounders of the exposure–mediator, mediator–outcome, and exposure–outcome effects: age, sex, *KCNE1*, *PITX2* and *MTHFR* (except in Model 2 and Model 3) polymorphisms, folate concentrations, and components identified in the principal components analysis: “Blood pressure”, “Diabetes”, “BMI-CRP”, and “Lipid”. ‡ There was no exposure (tHcy)*mediator (Renal-BNP) interaction in any model (all *p*-values > 0.400).

**Table 6:** Summary of the adjusted † mediation analysis to estimate reverse association between plasma homocysteine (tHcy) and non-valvular atrial fibrillation (NVAF): *PITX2 C>T* variant allele is the exposure, NVAF is the mediator, and ln(tHcy) is the outcome. Shown are results of the causal mediation analysis with (Model 1) and without (Model 2) Renal-BNP from the principal components analysis as a confounder. Corrected refers to relative risks estimated with a correction for a hypothetical non-differential error in measurement of tHcy.

	GMR (95%CI)	E-Value	Corrected
Model 1 ‡ (Renal-BNP as confounder)			
Pure natural direct effect (PNDE)	1.003 (0.965, 1.044)	---	1.002
Total natural direct effect (TNDE)	1.006 (0.973, 1.058)	---	1.005
Pure natural indirect effect (PNIE)	1.003 (0.998, 1.014)	---	1.002
Total natural indirect effect (TNIE)	1.006 (0.998, 1.021)	---	1.005
Total effect	1.009 (0.979, 1.068)	---	1.007
Model 2 ‡ (Renal-BNP not in the model)			
Pure natural direct effect (PNDE)	1.002 (0.867–1.170)	---	1.002
Total natural direct effect (TNDE)	1.004 (0.997–1.026)	---	1.004
Pure natural indirect effect (PNIE)	1.006 (0.998–1.026)	---	1.007
Total natural indirect effect (TNIE)	1.008 (0.998–1.027)	---	1.008
Total effect	1.010 (0.980–1.074)	---	1.010

† Variables included in all models as confounders of the exposure–mediator, mediator–outcome, and exposure–outcome effects: age, sex, *KCNE1* and *MTHFR* polymorphisms, folate concentrations, and components identified in the principal components analysis: “Blood pressure”, “Diabetes”, “BMI-CRP”, and “Lipid”. ‡ There was no exposure (*PITX2*)*mediator (NVAF) interaction in any model (all *p*-values > 0.500).

**Table 7:** Summary of the one-sample Mendelian randomization/instrumental variable analysis to estimate forward association between total plasma homocysteine (tHcy) and non-valvular atrial fibrillation (NVAF) (Model 1: *MTHFR 677C>T* variant allele is instrument, tHcy is exposure) and to estimate reverse association between tHcy and NVAF (Model 2: *PITX2 C>T* variant allele is instrument, NVAF is exposure, and ln[tHcy] is outcome). Effects are relative risks (RR) in Model 1 and geometric means ratios (GMR) in Model 2 with 95% confidence intervals.

	Effect (RR or GMR)	E-Value
Model 1: Forward association tHcy-NVAF (*MTHFR 677C>T* instrument, tHcy exposure, and NVAF outcome)		
*Effect of exposure on outcome*		
Ln(total plasma homocysteine)	2.333 (1.063–5.120)	4.100
*Effects of covariates (confounders) on outcome*		
*PITX2 C>T* variant allele	1.492 (1.158–1.920)	2.350
Male sex	1.890 (1.454–2.457)	3.190
“Lipid” (from principal components analysis)	0.867 (0.780–0.963)	0.637
Model 2: Reverse association tHcy-NVAF (*PITX2 C>T* instrument, NVAF exposure, and ln[tHcy] outcome)		
*Effect of exposure on outcome*		
NVAF	1.045 (0.573–1.907)	---
*Effects of covariates (confounders) on outcome*		
*MTHFR 677 C>T* variant allele	1.080 (1.018–1.147)	1.380
*KCNE1 112A>G* variant allele	1.043 (0.965–1.126)	---
Blood pressure” from principal components analysis	1.032 (0.924–1.154)	---
“BMI-CRP” from principal components analysis	1.020 (0.987–1.054)	---

## Data Availability

The original contributions presented in this study are included in the article/Supplementary Materials. Further inquiries can be directed to the corresponding author.

## Supplementary Materials

The following supporting information can be downloaded at: https://www.mdpi.com/article/10.3390/diagnostics15222870/s1.

## Abbreviations

The following abbreviations are used in this manuscript:

tHcy	homocysteine
AF	atrial fibrillation
NVAF	non-valvular AF
MR	Mendelian randomization
NTproBNP	N-terminal pro-B-type natriuretic peptide
GWAS	genome-wide association studies
MTHFR	methylenetetrahydrofolate reductase
SNPs	single nucleotide polymorphisms
I_Ks_	slowly activating delayed rectifying potassium current
BMI	body mass index
DNA	deoxyribonucleic acid
PCR	polymerase chain reaction
MR/IV	Mendelian randomization/instrumental variable
CRP	C-reactive protein
LDL-C	low-density lipoprotein cholesterol
PCA	principal components analysis
OR	odds ratios
RR	relative risks
HWE	Hardy-Weinberg equilibrium
BP	blood pressure
MSCT	multislice computed tomography
BNP	brain natriuretic peptide
Ln	natural logarithm of a number
Renal-BNP	creatinine, urea, NT-proBNP
Lipid	LDL-C, triglycerides
BMI-CRP	body mass index category, CRP and current smoking
GMR	geometric means ratios
PNIE	pure natural indirect effect
TNIE	pure total indirect effect
PNDE	pure natural direct effect
TNDE	total natural direct effect
CAD	coronary artery disease
RCTs	randomized trials
CVD	cardio/cerebrovascular disease
NIE	natural indirect effect
