# Supplementary material for: Common Methylenetetrahydrofolate Reductase Polymorphism MTHFR 677C>T (rs1801133), Plasma Homocysteine, and Non-Valvular Atrial Fibrillation in Overweight/Obese Patients: Causality Indicated by Mediation and One-Sample Mendelian Randomization Analysis
Source: Diagnostics (Basel). 2025 Nov 12;15(22):2870. doi: 10.3390/diagnostics15222870 (PMC12651033; doi:10.3390/diagnostics15222870)
Supplement: Supplementary file 1 [file diagnostics-15-02870-s001.zip › diagnostics-3921087-supplementary.pdf]

diagnostics

MDPI

Supplementary Material A. Supplemental methods

Contents

A.1. Outline of the Data Analysis Procedures
A.2. Conventional Case-Control Analysis
A.2.1. Overview
A.2.2. Methods for Covariate Adjustments/Control of Confounding
A.2.3. Sensitivity to Residual Confounding and Other Biases
A.3. Mediation and One-Sample MR/IV Analysis
A.3.1. Overview
A.3.2. Mediation Analysis
A.3.3. One-Sample MR/IV Analysis
A.4. Sample Size Considerations

A.1. Outline of the Data Analysis Procedures

We considered that no single analysis method per se was sufficient to reasonably support or reject the hypothesis about the causal role of tHcy in NVAF but that it should be addressed through several complementary procedures. We first implemented conventional analysis of a case–control study with four exposures successively considered as those of primary interest: (i) *MTHFR 677C>T* SNP is the exposure. As in previous similar studies, we expected that the analysis would fail to identify the association between the polymorphism and NVAF but considered it important as an example of the inadequacy of the approach; (ii) tHcy is the exposure. In line with similar previous studies, we expected association between tHcy and NVAF, possibly depending on covariate adjustment; (iii) *PITX2 C>T* SNP is the exposure. In line with the proven causal role of this SNP in AF, we expected to demonstrate its associations with NVAF. The SNP was considered as (a) a covariate in other analyses and (b) a tool for the assessment of reverse association between tHcy and NVAF; (iv) *KCNE1 112A>G* SNP is the exposure. We considered it a potentially relevant confounder, and a potential tool in the assessment of reverse association between tHcy and NVAF. In the second step, we conducted mediation and one-sample MR/IV analyses with the intention to asses (i) the association between the *MTHFR 677C>T* polymorphism and NVAF when tHcy is considered a mediator; (ii) association between tHcy and NVAF in mediation analysis (tHcy is exposure) and in MR/IV analysis (*MTHFR 677C>T* is instrument); (iii) reverse association between NVAF and tHcy using *PITX2 C>T* and/or *KCNE1 112A>G* as an exposure (mediation analysis) or as an instrument (MR/IV analysis), NVAF as a mediator (mediation analysis) or exposure (MR/IV analysis), and tHcy as the outcome.

For covariate adjustment, we considered plasma folate concentrations and a number of established conventional risk factors for AF [1–6]: age (older), sex (male), current smoking, obesity (vs. overweight), existing hypertension, actual blood pressure (systolic, diastolic) (higher), diabetes mellitus, C-reactive protein (higher), triglycerides (lower), LDL-C (lower), NT-proBNP (higher), creatinine, and urea (higher). Other classical risk factors (cardiac, renal, hepatic, systemic diseases/inflammation, malignancy, advanced COPD, and obstructive sleep apnea) were controlled by exclusion criteria. Since cases in

this study were prevalent NVAF patients, we considered that echocardiographic indices were more likely consequent to than causal to NVAF. Likewise, apart from the listed variables, standard laboratory indicators were considered subsumed within the demographic and comorbidity characteristics that we accounted for. All generated estimates were reported with 95% confidence intervals and all were submitted to the analysis of sensitivity to residual confounding and other biases.

A.2. Conventional Case–Control Analysis

A.2.1. Overview

Figure S1 depicts settings within which *MTFHR*, *PITX2*, and *KCNE1* SNPs and tHcy were evaluated for their association with NVAF in a conventional case–control type of analysis. Figure S1A (*MTHFR* SNP is exposure) and Figure S1B (tHcy is exposure) depict an approach commonly used in similar studies, which, however, is inappropriate, since it disregards the fact that tHcy is affected by the *MTHFR 677C>T* SNP (and is therefore a mediator and not a confounder), and that downstream to tHcy, there could be other mediators on a path between the *MTHFR* polymorphism and/or tHcy and the outcome. The settings with *PITX2* (Figure S1C) or *KCNE1* (Figure S1D) SNPs as exposures are adequately defined because current knowledge does not suggest that any of the depicted covariates should be viewed as a mediator of the "effect" of the exposure on the outcome. None of the listed covariates are, based on the existing knowledge, a likely collider on any of the assessed paths.

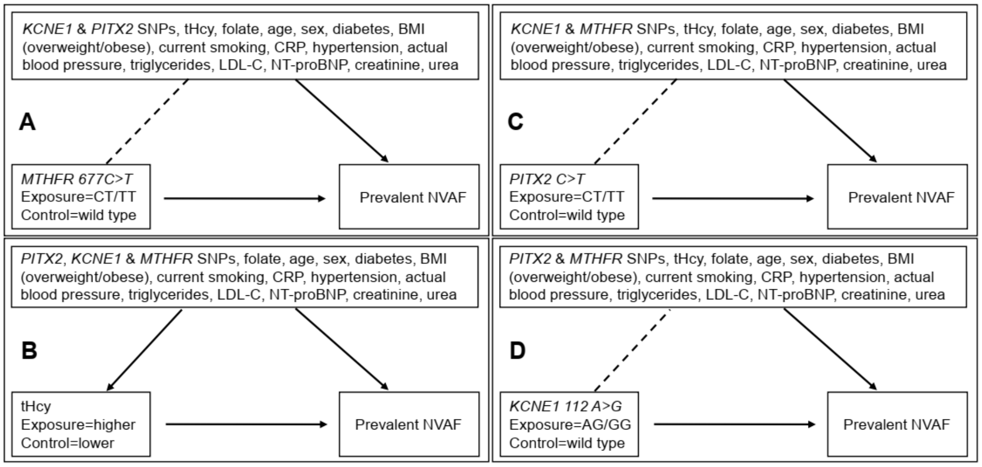

Figure S1. Schematic of directed acyclic graphs (DAGs) outlining settings to estimate association between exposures of interest [*MTHFR* SNP (**A**), tHcy (**B**), *PITX2* SNP (**C**) and *KCNE1* SNP (**D**), and prevalent NVAF in a case–control type of analysis. Directed full arrows indicate the estimated paths and possible confounder effects on the exposure (tHcy) and the outcome. Dashed lines indicate possible imbalances in potential confounders between levels of polymorphisms.

A.2.2. Methods for Covariate Adjustments/Control of Confounding

For a multivariable logistic model (typically used in conventional analysis of prevalent case–controls studies) to be meaningful, the number of covariates that we considered (Figure S1) would have required a considerably larger numbers of cases and controls than what was feasible to us over a reasonable period of time. We therefore used the following approach: (i) we first attempted to achieve a balance between cases and controls on as many covariates as possible (except the one that was being evaluated for its association with NVAF) using optimization-based weighting with the average treatment effect as the estimand.

Optimization-based weighting is one of a number of methods available to achieve balance between two or more groups with respect to their properties. It is specific in that it enables distributional balance and (where feasible, based on data characteristics in the groups to be balanced) generation of stable balancing weights by solving a constrained convex optimization problem [7,8]. In practice, the user provides balance constraints, i.e., for each characteristic intended to be balanced, one provides the preferred standardized mean difference (d) between the groups. Ideal balance implies that d = 0.000, but it is generally assumed that groups are well balanced for a given variable if d < 0.1, and if the solution is possible—given the data—the procedure yields stable weights that maximize the effective sample size (all weighting methods increase the variance, resulting in a reduced precision of the estimates in the balanced sets, as if the number of units in the weighted sample were reduced vs. the original sample). At the same time, the objective is to obtain mean weights within each group equal to 1.0, with a reasonably low standard deviation (i.e., reasonably narrow range of assigned weights). The procedure may also fail to find a solution to the problem or results in highly variable weights and a low effective sample size, in which case it does not achieve the intended purpose. Weighting was performed in package WeightIt [9] in R. (ii) Covariates that could not be adequately balanced at the same time with mild–moderate variability of the assigned weights and a limited increase in variance were excluded from the process. In the case of a larger number of covariates that remained unbalanced between cases and controls, we anticipated a dimension reduction using principal components analysis (PCA) for mixed categorical and continuous data [10] with varimax rotation and selection of dimensions with eigenverctors > 1.0 (implemented in R package PCAmixedata [11]). Generated components (i.e., their coefficients) were used as covariates in regression models fitted to weighted data. Mulitvariable (weighted) logistic regression models with robust standard error estimation were fitted in SAS for Windows 9.4 (SAS Inc., Cary, NC, USA). The result of interest was the estimated adjusted strength of association between the exposure analyzed in the respective model and NVAF, expressed as an odds ratio (OR). We also report the optimal approximations of relative risks (RRs) by conversion from odds ratios [12], since RRs are more intuitive for interpretation, particularly when considered together with indicators of susceptibility to residual confounding and other biases.

A.2.3. Sensitivity to Residual Confounding and Other Biases

All fully adjusted estimates that indicated associations between a particular exposure (SNP, tHcy) and the outcome (NVAF) were evaluated for sensitivity to (residual) confounding by generation of the E-value (package Evalue in R) [13]. The E-value is expressed on the relative risk (RR) scale and denotes the strength of association that a confounding set needs to have with the outcome and with the exposure in order to explain away the observed exposure–outcome association [13]. In addition to residual confounding, we considered that the present estimates might have been affected by bias due to misclassification of the outcome. Regarding the former, it should be noted that the present estimates were generated while accounting for a substantial number of potential confounders. However, a number of other, genetic or non-genetic, potential confounders likely remained unaddressed. To account for residual confounding, we assumed a hypothetical confounder (or a set of confounders) with a three-times-higher prevalence among variant allele carriers (or patients with "higher" homocysteine levels) (exposed) than among wild-type subjects (or patients with "average" homocysteine levels) (controls), e.g., 60% vs. 20%, that was strongly associated with AF expressed as RR = 1.50 (50% increase in probability of AF) or, conversely, RR = 1/1.5 = 0.67 (33% reduction in the probability of AF). Regarding misclassification bias, we considered that all the study participants with NVAF were correctly diagnosed. We also considered that all patients

who were free of AF (and therefore classified as "controls") at the time of study participation were also correctly classified. However, the possibility that some of the patients who were considered "controls" would have eventually developed AF at some point in their later life could not be excluded. Based on the estimates generated in the analysis of the Framingham heart study [14] and in the Danish population analysis [15], we considered it reasonable to assume a 30% lifetime risk of AF in the present patient sample. This implies that 30% of the current "controls" would eventually develop AF—in other words, some 30% of the current controls (n = 54) were misclassified as controls. In this respect, sensitivity of the present identification of (NV)AF was 77% (180/234), whereas specificity was 100%, and these values were applicable to both variant carriers and wild-type subjects regarding each of the tested exposures of interest. For each of the tested SNPs and tHcy, we used fully adjusted estimated probabilities of NVAF for variant carriers and wild-type subjects (i.e., patients with homocysteine at mean vs. at 33% higher than mean) to correct the generated estimates for these multiple biases [16]. We used the package episensr in R [17].

A.3. Mediation and One-Sample MR/IV Analysis

A.3.1. Overview

The planned mediation and one-sample MR/IV analyses depended on the assumption that the *MTHFR 677C>T* SNP affected plasma tHcy concentrations. Therefore, we first assessed the impact of the polymorphism on tHcy overall and separately in cases and in controls: we estimated raw and adjusted differences in tHcy between different genotypes (linear models fitted to ln-transformed tHcy [SAS for Windows 9.4, SAS Inc. Cary, NC, USA], with key factors known to affect tHcy concentrations [18]—age, sex, current smoking, and folate concentrations—as covariates). Next, we repeated the PCA (as described in relation to the case–control analysis) to reduce the dimensionality of covariates: considering their roles in the planned analyses, as well as their known or likely impact on the outcome (NVAF) and/or on tHcy, *MTHFR*, *PITX2* and *KCNE1* SNPs, age, sex, and folate concentrations, were considered as individual variables, while the remaining ones (LDL-C, trigylcerides, fasting glucose and diabetes, blood pressure and diagnosis of hypertension, CRP, NT-proBNP, urea, creatinine, body mass index category, and current smoking) were included in the PCA. We then conducted the mediation and one-sample MR/IV analyses to (i) assess forward associations between *MTHFR 677C>T* [exposure (in mediation) or instrument (in MR/IV)] and/or tHcy (exposure or mediator) and NVAF; (ii) assess reverse tHcy-NVAF association using *PITX2* as the exposure (in mediation) or instrument (in MR/IV), NVAF as the mediator (in mediation) or exposure (in MR/IV), and tHcy as the outcome.

A.3.2. Mediation Analysis

We implemented causal (potential outcomes) and traditional mediation analysis. Although conceptually and computationally different, the two methods address the same problem and yield corresponding quantities. While causal mediation is preferred nowadays, the traditional approach has elaborated methods for multiple mediators and can thus contribute complementary information [19,20].

Causal mediation: This method operates within the contrafactual (potential outcomes) framework. It enables the identification of five "effects" of interest in understanding the link between an exposure and the outcome [19,20]:

Pure natural direct effect (PNDE) is the non-mediated part of the effect of exposure on the outcome: to estimate it, the effect of the mediator (the mediated part) is blocked by fixing the mediator value in all subjects at the value it would have taken had all the subjects been at the control level of the exposure (e.g., with the *MTHFR* polymorphism as

an exposure and tHcy as a mediator, as if all were *MTHFR* wild-type subjects [with a "lower" tHcy level]). PNDE is formally defined as a difference in potential outcomes assuming that all subjects are exposed (e.g., MTHFR variant carriers) vs. assuming that all are non-exposed (e.g., MTHFR wild type), while the mediator (tHcy) is, in all subjects, fixed at the "non-exposed" level:

$$PNDE = E[Y(1, M(0))] - E[Y(0, M(0))]$$

Total natural direct effect (TNDE) is the non-mediated part of the effect of exposure on the outcome where the mediated part is blocked by being fixed, in all subjects, at the value it would have taken had all the subjects been at the active level of the exposure (e.g., as if all were *MTHFR* variant carriers [with a "higher" tHcy level]). PNDE is formally defined as a difference in potential outcomes assuming that all subjects are exposed (e.g., *MTHFR* variant carriers) vs. assuming that all are non-exposed (e.g., *MTHFR* wild type), while the mediator (tHcy) is, in all subjects, fixed at the "exposed" level:

$$TNDE = E[Y(1, M(1))] - E[Y(0, M(1))]$$

Pure natural indirect effect (PNIE) is the part of the effect of exposure on the outcome that is transmitted through a mediator (mediated), that is, an effect due to the change in mediator induced by the exposure. To estimate it, the direct effect of the exposure is blocked by being fixed in all subjects at the control level—with *MTHFR* SNP as an exposure, PNIE quantifies the effect of the mediator on the outcome assuming that all subjects are *MTHFR* wild type. PNIE is formally defined as a difference in potential outcomes assuming that all subjects have mediator values as if exposed (e.g., *MTHFR* variant carriers [i.e., "higher" tHcy]) vs. assuming that all have mediator values as if non-exposed (e.g., *MTHFR* wild type [i.e., "lower" tHcy]), while the exposure is, in all subjects, fixed at the "non-exposed" level:

$$PNIE = E[Y(0, M(1))] - E[Y(0, M(0))]$$

Total nautral indirect effect (TNIE) is the part of the effect of exposure on the outcome that is transmitted through the mediator: to estimate it, the direct effect of the exposure is blocked by being fixed at the exposed level—with *MTHFR* SNP as an exposure, TNIE quantifies the effect of the mediator on the outcome assuming that all subjects are *MTHFR* variant carriers. TNIE is formally defined as a difference in potential outcomes assuming that all subjects have mediator values as if exposed (e.g., *MTHFR* variant carriers [i.e., "higher" tHcy]) vs. assuming that all have mediator values as if non-exposed (e.g., *MTHFR* wild type [i.e., "lower" tHcy]), while the exposure is, in all subjects, fixed at the "exposed" level:

$$TNIE = E[Y(1, M(1))] - E[Y(1, M(0))]$$

Total effect (TE) is the total effect of the exposure on the outcome, i.e., combined direct and indirect. In other words, it is a difference between potential outcomes under the active level of exposure + active level of the mediator in all subjects and potential outcomes under the control level of exposure and control level of the mediator in all subjects. TE is formally defined as follows:

$$TE = E[Y(1, M(1))] - E[Y(0, M(0))]$$

Causal mediation was implemented in package CMAverse [21] in R and, as recommended [19], all models included an exposure–mediator interaction. All estimates were evaluated for susceptibility to unmeasured confounding by the generation of E-values and were also corrected for the effect of a hypothetical nondifferential measurement error of tHcy, whether considered as a mediator or as an exposure [19,21].

We assumed an error corresponding to 1/6 of the standard deviation of the ln-transformed homocysteine concentrations.

Traditional mediation analysis is based on the concept of structural equation modeling (SEM). It enables the identification of total, direct and indirect effects, and the inclusion of multiple mediators [22]. It was implemented in SAS 9.4 for Windows using macro Process [22].

A.3.3. One-Sample MR/IV Analysis

One-sample MR/IV analysis uses genetic variants as instruments to assess causal relationships between exposures and outcomes under the known conditions of the Mendelian randomization concept (instrument is associated with exposure, affects the outcome only through the effect on exposure, and is not associated with the outcome due to confounding paths) and enables the inclusion of covariates [23]. The covariates (i.e., potential confounders) need to be limited in number (avoid overpatametrization) and carefully selected—they should not be confused for mediators or colliders [23]. We intended to use this approach to complement the mediation analysis. For the estimation of the forward association between tHcy (exposure) and NVAF (outcome), we planned a model with *MTHFR 677C>T* polimorphsm as the instrument (dichotomized to variant allele vs. wild type) and the inclusion of covariates based on the imbalance in characteristics between variant allele carriers and wild-type subjects. The *MTHFR 677C>T* SNP has been linked to other phenotypic characteristics in GWASs besides tHcy: (i) association has been reported with blood pressure/hypertension [24]; (ii) serine levels (inverse association), mean erythrocyte volume, and mean erythrocyte hemoglobin concentration [24]. In this respect, this SNP could be viewed as pleiotropic (horizontal pleiotropy) and, as such, not an adequate instrument in MR analysis for evaluation of the effect of tHcy on an outcome [23]. However, the GWAS association with blood pressure/hypertension has been contradicted, and an MR study indicated no causal effect of tHcy on hypertension [25,26]. As for the other GWAS-associated phenotypic traits, we consider that they could hardly be understood in a different way but as downstream conseqences of the effect of the SNP on tHcy (i.e., MCV, MCHC, and serine levels). This would correspond to a phenomenon of vertical pleiotropy, where the SNP is the instrument, tHcy is the exposure, and other traits are mediators of the exposure effect on the outcome [23]. Moreover, this polymorphism has been used to demonstrate the causal effects of tHcy on the risk of small vessel ischemic stroke [27,28]. We therefore considered it justified to employ the MR analysis as an additional tool to evaluate the possibility that tHcy had a causal effect on NVAF. The analysis was based on the multiplicative structural mean model with a generalized method of moments estimation, as implemented in package OneSampleMR [29] in R. The procedure estimates causal risk ratios as measures of association (effect) of the exposure and the covariates on the outcome. For assessment of a potential reverse association between tHcy and NVAF, we planned to estimate a model with *PITX2* C>T SNP as the instrument (dichotomized to variant allele carriage vs. wild type), NVAF as the exposure and ln(tHcy) as the outcome, with selected covariates based on imbalances between *PITX2* variant carriers and wild-type subjects. The analysis was based on two-stage predictor substitution estimators, as implemented in package OneSampleMR [29]. The method first includes regression of the exposure (NVAF) on the instrument and covariates (confounders) that generates predicted values of the exposure. The second stage model is then fitted, in which the outcome is regressed on the predicted values of the exposure (and covariates). The procedure estimates mean differences as measures of association (effect) between the exposure and the covariates and the outcome. Since the outcome is ln-transformed, the differences are eventually expressed as geometric means ratios (GMRs).

A.4. Sample Size Considerations

We intended to enroll numbers of cases and controls sufficient to detect (replicate) the strength of the association between *PITX2 C>T* [30], *KCNE1 112A>G* in Caucasians [31], or tHcy [32] and (NV)AF previously reported in prevalent case–control studies. No particular calculations were performed regarding mediation or MR/IV analyses. The assumptions were that (i) the study is unmatched case–control study; (ii) the number of cases and controls is equal; (iii) the increase in variance due to covariate balancing is such that the effective sample size is not more than 10% lower than the actual number of subjects, comparably for the cases and controls; (iv) the prevalence of exposure (variant allele carriers) among controls may vary between 35% and 65%; (v) the minimum detectable strength of association between an exposure and the case status is expressed as an adjusted OR = 1.90 (or 0.53) (corresponds to prevalence ratio of approximately 1.38, i.e., 0.73; absolute adjusted difference in exposure between cases and controls is around 11% to 14%); (vi) the exposure of interest is not correlated with covariates in a logistic model; and (vii) the desired power is 80% at two-sided alpha = 0.05. The required total number of subjects under the above conditions was calculated to be ~340 [33]. We therefore aimed to enroll around 185 cases and 185 controls.

References

1. Hindricks G, Potpara T, Dagres N, et al. 2020 ESC Guidelines for the diagnosis and management of atrial fibrillation developed in collarboratino with European association of cardio-thoracic surgery (EACTS). Eur Heart J 2021, 42, 373-498.
2. Wang N, Yu Y, Sun Y, et al. Acquired risk factor and incident atrial fibrillation according to age and genetic predisposition. Eur Heart J 2023, 44, 4982-4993.
3. Wang J, Jiang C, Li S, et al. Systolic blood pressure time in target range and incident atrial fibrillation in patients with hypertension: insight from the SPRINT trial. Hypertension 2023, 80, 2306-2314.
4. Young LJ, Antwi-Boasiako S, Ferrall J. Wold LE, Mohler PJ, El Refaey M. Genetic and non-genetic risk factors associated with atrial fibrillation. Life Sci 2022, 299, 120529. https://doi.org.10.1016/j.lfs.2022.120529
5. Elliott AD, Middeldorp ME, Van Gelder IC, Albert CM, Sanders P. Author Correction: Epidemiology and modifiable risk factors for atrial fibrillation. Nat Rev Cardiol 2023, 20, 429.
6. Schnabel RB, Larson MG, Yamamoto JF, et al. Relations of biomarkers of distinct pathophysiological pathways and atrial fibrillation incidence in the community. Circulation 2010, 121, 200-207.
7. Zubizarreta JR. Stable weights that balance covariates for estimation with incomplete outcome data. J Am Stat Assoc 2015, 110, 910-922.
8. Wang Y, Zubizarreta JR. Minimal dispersion approximately balancing weights: asymptotic properties and practical considerations. Biometrika 2020, 107, 93-105.
9. Greifer N. WeightIt: Weighting for covariate balance in observational studies. R package version 1.4.0.9001, 2025, https://ngreifer.github.io/WeightIt/
10. Kiers HA. Simple structure in component analysis techniques for mixtures of qualitative and quantitative variables. Psychometrika 1991, 56, 197-212.
11. Chavent M, Kuentz V, Labenne A, Saracco J. Multivariate analysis of mixed data. The R package PCAmixdata. 2022, arXiv 2022, 1411.4911
12. VanderWeele T. Optimal approximate converstions of odds ratios and hazard ratios to risk ratios. Biometrics 2020, 76, 746-752.
13. VanderWeele T, Ding P. Sensitivity analysis in observational research: Introducting the E-value. Ann Intern Med 2017, 104, 291-302.

14. Staerk L, Wang B, Preis SR, et al. Lifetime risk of atrial fibrillation according to optimal, borderline, or elevated levels of risk factors: cohort study based on longitudinal data from the Framingham Heart Study. BMJ 2018, 361, k1453.
15. Vinter N, Cordsen P, Johsnen SP, et al. Temporal trends in lifetime risks of atrial fibrillation and its complications between 2000 and 2022: Danish nationwide population based cohort study. BMJ 2024, 385, e077209.
16. Fox MP, MacLehose RF, Lash TL. Applying quantitative bias analysis to epidemiologic data. 2nd ed., Cham: Springer 2021.
17. Haine D. The episensr package: basic sensitivity analysis of epidemiological results. doi:10.5281/zenodo.8299430, R package version 1.3.0, 2023. https://dhaine.codeberg.page/episensr/.
18. McCaddon A, Miller JW. Homocysteine – a retrospective and prospective appraisal. Front Nutrition 2023, 10:1179807. https://doi.org/10.3389/fnut.2023.1179807
19. VanderWeele TJ. Explanation in causal inference. Methods for mediation and interaction. New York, NJ: Oxford University Press 2015.
20. Rijnhart JJM, Valente MJ, MacKinnon DP, Twisk JWR, Heymans M. The use of traditional and causal estimators for mediation models with a binary outcome and exposure-mediator interaction. Struct Equat Modeling Multidis J 2021, 38, 345-355.
21. Shi B, Choirat C, Coull BA, VanderWeele TJ, Valeri L. CMAverse: a suite of functions for reproducible causal mediation analysis. Epidemiology 2021, 32, e20-e22.
22. Hayes AF.Introduction to mediation, moderation, and conditional process analysis. New York, NJ:The Guilford press 2013.
23. Burgess S, Davey Smith G, Davies NM, et al. Guidelines for performing Mendelian randomization investigations: update for summer 2023, Wellcome Open Res 2023, 4, 186. https://doi.org/10.12688/wellcomeopenres.15555.3
24. GWAS Catalog. The NHGRI-EBI Catalog of human genom-wide association studies. Available at: GWAS Catalog, Accessed May 21, 2025.
25. Meng H, Huang S, Yang Y, He X, Fei L, Xing Y. Association between MTHFR polymorphisms and the risk of essential hypertension: an updated meta-analysis. Front Genet 2021, 12, 698590. doi: 10.3389/fgene.2021.698590
26. Borges MC, Hartwig FP, Oliveira IO, Horta BL. Is there a causal role of homocysteine in blood pressure? A mendelian randomization study. Am J Clin Nutrition 2016, 103, 39-49.
27. Rutten-Jacobs LCA, Traylor M, Adib-Samii P, Thijs V, Sudlow C, Rothwell PM. Association of MTHFR C677T genotype with ischemic stroke is confined to cerebral small vessel disease subtype. Stroke 2016, 47, 646-651.
28. Wang M, Daghlas I, Zhang Z, Gill D, Liu D. MTFHR polymorphisms, homocysteine elevation, and ischemic stroke susceptibility in East Asian and European populations. Neurology 2025, 104, 3, e210245. https://doi.org/10.1212/WNL.0000000000210245
29. Palmer T, Spiller W, Sanderson E. OneSampleMR: One sample Mendelian randomization and instrumental variable analyses. MRC Integrative Epidemiology Unit, University of Bristol, Bristol UK, 2023, https://remlapmot.github.io/OneSampleMR/.
30. Putra RH, Dharmadjati BB, Pikis BS, Maghfirah I, Isaridha IA, Arnindita JN. Five single-nucleotide polymorphisms in the PITX2 gene as risk factor for atrial fibrillation. Folia Med Indones 2023, 59, 85-91.
31. Jiang YF, Chen M, Zhang NN, et al. Association between KCNE1 G38S gene polymorphism and risk of atrial fibrillation. Medicine 2017, 96, e7253. https://doi.org/10.1097/MD.0000000000007253

32. Giusti B, Gori AM, Marcucci R, et al. Role of C677T and A1298C MTHFR, A2756G MTR and -786 C/T eNOS gene polymoprhisms in atrial fibrillation susceptibility. PlosONE 2007, 2, e495. doi:10.1371/journal.pone.0000495
33. Gail MM, Haneuse S. Power and sample size for case-control studies. In : Borgan O, Breslow NE, Chatterjee N, Gail MH, Scott A, Wild CJ, eds. Handbook of statistical methods for case-control studies. Boca Raton, FL: Taylor&Francis Group, 163-188.

Supplementary Material B. Additional Results I: Patient Characteristics

Contents

Figure S2. Relationship between plasma homocysteine and folate concentrations.

Table S1. Characteristics of cases and controls: blood cell counts, clinical chemistry, and biochemistry.

Table S2. Characteristics of cases and controls: echocardiography.

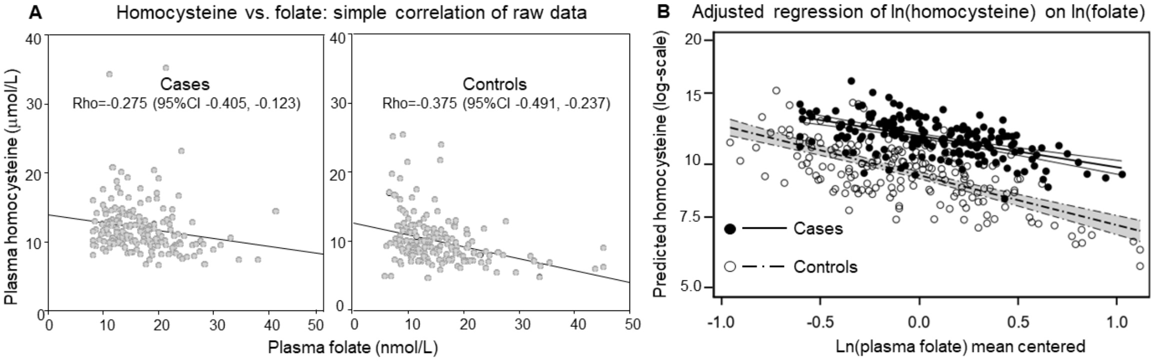

Figure S2. Relationship between plasma homocysteine and plasma folate concentrations. (**A**) Simple correlation between plasma homocysteine and plasma folate concentrations, separately in cases (patients with NVAF) and in controls (subjects without AF). Inserted are Spearman's rank correlation coefficients. (**B**) Adjusted regression of ln(homocysteine) on ln(folate) in cases and controls. A general linear model (restricted maximum likelihood estimation) was fitted to ln(homocysteine) with fixed effects: ln(folate) (mean centered), case status, ln(folate)*case status interaction, age, sex, current smoking, and *MTHFR 677C>T* (dichotomized as variant carriers or wild-type subjects). Changes in plasma homocysteine associated with 33% increase in folate concentration are expressed as geometric means ratios (GMRs) and were similar in cases (GMR = 0.96, 95%CI 0.93–0.99) (i.e., 4% reduction by 33% increase in folate) and in controls (GMR = 0.92, 95%CI 0.90–0.95) (8% reduction by 33% increase in folate) (the difference between cases and controls = −4.0%, 95%CI −9.0 to 1.0). Note, however, that the homocysteine concentrations in cases were "shifted" towards higher values as compared to the controls.

Table S1. Characteristics of cases and controls: blood cell counts, clinical chemistry, and biochemistry. Data are median (quartiles) and standardized mean differences (d) between cases and controls.

	All	Cases	Controls	d
N	359	180	179	---
Red blood cells ($\times 10^9$/L)	4.8 (4.6–5.1)	4.9 (4.5–5.1)	4.8 (4.6–5.0)	0.107
MCV (fL)	88 (86–90)	88 (86–90)	88 (85–90)	0.083

MCHC (g/L)	332 (324–340)	333 (327–343)	328 (321–338)	0.478
MCH (pg)	29 (28–30)	30 (29–31)	29 (28–30)	0.368
Hemoglobin (g/L)	142 (135–152)	146 (137–154)	140 (132–146)	0.403
Red cell distribution width (%)	13.2 (12.6–13.8)	13.4 (12.8–14.0)	13.1 (12.5–13.5)	0.371
White blood cells (×10⁹/L)	6.6 (5.6–8.1)	6.5 (5.6–8.0)	6.9 (5.6–8.2)	−0.003
Platelets (×10⁹/L)	243 (208–284)	233 (198–261)	253 (230–297)	−0.588
Fasting glucose (mmol/L)	5.6 (5.2–6.0)	5.7 (5.3–6.1)	5.5 (5.2–5.9)	0.118
Sodium (mmol/L)	140 (138–141)	141 (139–142)	139 (138–140)	0.792
Potassium (mmol/L)	4.2 (3.9–4.4)	4.2 (4.0–4.5)	4.1 (3.9–4.3)	0.456
Chloride (mmol/L)	109 (107–110)	109 (108–110)	108 (104–109)	0.508
Aspartate transaminase (U/L)	25 (20–31)	27 (22.32)	25 (19–29)	0.193
Alanine transaminase (U/L)	27 (19–34)	29 (24–35)	24 (17–32)	0.307
International normalized ratio	1.03 (0.98–1.12)	1.05 (1.00–1.14)	1.01 (0.93–1.10)	0.515
Alkaline phosphatase (U/L)	62 (53–72)	63 (56–79)	61 (51–71)	0.467
Lactate dehydrogenase (U/L)	182 (165–196)	193 (178–203)	171 (156–182)	0.886
D-dimer (mg/L)	0.26 (0.19–0.55)	0.30 (0.19–0.56)	0.26 (0.19–0.55)	0.256
Total cholesterol (mmol/L)	5.5 (4.7–6.1)	5.3 (4.3–5.9)	5.7 (4.9–6.1)	−0.401
HDL cholesterol (mmol/L)	1.2 (1.1–1.4)	1.2 (1.0–1.4)	1.2 (1.1–1.5)	−0.014

HDL—high-density lipoprotein; MCV—mean corpuscular volume; MCHC—mean corpuscular hemoglobin concentration; and MCH—mean corpuscular hemoglobin.

Table S2. Characteristics of cases and controls: echocardiography. Data are count (%), median (quartiles), and standardized mean differences between cases and controls (d).

	All	Cases	Controls	d
N	359	180	179	---
LV internal diameter end-diastole(mm)	51 (48–54)	53 (49–55)	50 (48–52)	0.614
LV internal diameter end-systole (mm)	34 (32–36)	34 (31–36)	35 (32–36)	−0.108
Intraventricular septal end-diastole (mm)	11 (10–12)	12 (11–13)	10 (9–12)	0.778
LV posterior wall end-diastole (mm)	10 (9–12)	11 (10–12)	10 (9–11)	0.525
Contractility				
No segmental aberrations	341 (95.0)	162 (90.0)	179 (100)	−0.471
Hypokinesia	18 (5.0)	18 (10.0)	0	0.471
Left ventricle ejection fraction (%)	65 (60–67)	62 (57–66)	66 (65–68)	−0.950
Left ventricle fractional shortening (%)	36 (32–37)	32 (28–36)	36 (35–38)	−1.069
Diastolic dysfunction				
None (normal diastole)	74 (20.6)	7 (3.9)	67 (37.4)	−0.910
Stage 1	185 (51.5)	83 (46.1)	102 (57.0)	−0.219
Stage 2	60 (16.7)	50 (27.8)	5 (5.6)	0.624
Stage 3	1 (0.3)	1 (0.6)	0	0.106
Stage 4	0	0	0	---
Not assessable—atrial fibrillation	39 (10.0)	39 (21.7)	0	0.744
Right ventricle end-diastolic diameter (mm)	31 (29–33)	32 (30–33)	30 (28–32)	0.648
Tricuspid annular plane syst. excursion (mm)	20 (19–22)	20 (19–22)	21 (20–22)	−0.504
Left atrial volume (mL)	65 (48–81)	76 (65–92)	49 (43–64)	1.313
Left atrium diameter PLAX (cm)	4.2 (3.7–4.8)	4.6 (4.2–5.2)	3.8 (3.5–4.2)	0.459
Right atrial volume (mL)	45 (38–55)	52 (45–63)	38 (32–44)	1.271
Aortic root (mm)	33 (31–35)	34 (33–36)	32 (30–34)	0.829
Aortic valve maximal velocity (ms)	1.6 (1.1–18)	1.7 (1.5–1.8)	1.5 (1.4–1.7)	0.052
Aortic regurgitation				
None or trace	225 (62.7)	79 (43.9)	146 (81.6)	−0.846
Mild	121 (33.7)	92 (51.1)	29 (16.2)	0.795
Moderate	13 (3.6)	9 (5.0)	4 (2.2)	0.149

Mitral regurgitation				
None or trace	137 (38.2)	16 (8.9)	121 (67.7)	−1.516
Mild	151 (42.1)	107 (59.4)	44 (24.6)	0.755
Moderate	64 (17.8)	50 (27.8)	14 (7.8)	0.540
Moderate–severe	7 (1.9)	7 (3.9)	0	0.285
Tricuspid regurgitation				
None or trace	219 (61.0)	86 (47.8)	133 (74.3)	−0.565
Mild	120 (33.4)	77 (42.8)	43 (24.0)	0.406
Moderate	18 (5.0)	15 (8.3)	3 (1.7)	0.309
Moderate–severe	2 (0.6)	2 (1.1)	0	0.150
Aortic stenosis				
None	333 (92.8)	165 (91.7))	168 (93.9)	−0.085
Mild	24 (6.7)	13 (7.2)	11 (6.1)	0.043
Moderate	1 (0.3)	1 (0.6)	0	0.106
Severe	1 (0.3)	1 (0.6)	0	0.106
Mitral stenosis	0	0	0	---
Tricuspid stenosis	0	0	0	---
Transaortic pressure gradient (mmHg)	15 (12–20)	18 (14–25)	13 (10–16)	1.012
Pulmonary artery pressure syst. (mmHg)	20 (17–30)	25 (20–35)	18 (15–21)	1.139
Pulmonary velocity acceleration time (ms)	114 (100–128)	106 (92–120)	122 (112–134)	−0.975

LV—left ventricle; PLAX—parasternal long axis.

Supplementary Material C. Additional Results II: Results of Optimization-Based Weighting, of Dimension Reduction in Unbalanced Covariates, and Sensitivity of the Adjusted Estimates to Bias

Contents

C.1. Results of optimization-based weighting for conventional case–control analysis.

Table S3. For estimation of association between *MTHFR 677C>T* and NVAF.

Table S4. For estimation of association between tHcy and NVAF.

Table S5. For estimation of association between *PITX2 C>T* and NVAF.

Table S6. For estimation of association between *KCNE1 112A>G* and NVAF.

C.2. Principal components analysis to reduce dimensionality of covariates that could not be adequately balanced (Figure S3).

C.3. Partial correlation analysis of covariates (Table S7).

C.4. Bias-corrected estimates (Table S8).

C.1. Results of optimization-based weighting for conventional case–control analysis

To estimate associations between the prevalent NVAF and *MTHFR 677C>T* SNP, tHcy, and *PITX2 C>T* and *KCNE1 112A>G* SNPs, optimization-based weighting was implemented four times, once for each of these four exposures—balancing was attempted on all other variables: other three characteristics of interest, age, sex, body mass index, diabetes, folate concentrations, current smoking, existing diagnosis of hypertension, actual blood pressure (systolic, diastolic), serum urea and creatinine, NT-proBNP, CRP, LDL-C, and triglyceride concentrations. As depicted in subsequent tables (Tables S3–S6),

in all procedures adequate balance (d = 0.000, mild variability of the assigned weights, effective sample size by <10% lower than the actual number of subjects) was achieved regarding SNPs, folate concentrations, current smoking, diabetes, and body mass index categories (overweight or obese). Age, sex, tHcy (when a covariate), pre-existing hypertension, actual blood pressure, NT-proBNP, CRP, serum urea and creatinine, triglyceride, and LDL-C concentrations differed between cases and controls to the extent that precluded adequate balancing—hence, these variables remained clearly imbalanced between the cases and controls after weighting.

Table S3. Characteristics of cases and controls after covariate balancing undertaken to estimate the association between *MTHFR677 C>T* (rs1801133) [1] polymorphism and NVAF. Data are weighted percentages, or mean ± standard deviation, or geometric mean (%coefficient of variation) for ln-transformed variables. Standardized mean differences (d) < 0.1 indicate irrelevant differences.

	**Cases**	**Controls**	**d**
N	180	179	---
Variables used for balancing			
KCNE1 A>G (rs1805127)			
AA	22.0	22.0	0.000
AG	47.6	47.6	0.000
GG	30.4	30.4	0.000
PITX2 C>T (2200733)			
CC	63.5	63.5	0.000
CT	32.0	32.0	0.000
TT	4.5	4.5	0.000
Diabetes	17.5	17.5	0.000
Body mass index category			
Overweight	55.2	55.2	0.000
Obese	44.8	44.8	0.000
Ln(folate concentration)	14.8 (33.6)	14.8 (43.0)	0.000
Current smoking	17.6	17.6	0.000
Exposure of primary interest			
MTHFR 677C>T (rs1801133) variant carrier	59.0	53.5	0.111
MTHFR 677C>T (rs1801133) wild type	41.0	46.5	−0.111
Covariates that could not be balanced			
Age	64 ± 10	49 ± 13	1.376
Men	66.6	34.2	0.681
Hypertension	92.1	48.2	1.091
Systolic blood pressure	147 ± 19	127 ± 12	1.230
Diastolic blood pressure	88 ± 10	78 ± 8	1.054
Ln(homocysteine concentration)	11.7 (26.2)	9.4 (31.2)	0.765
Ln(creatinine concentration)	88 (24)	73 (21)	0.857
Ln(urea concentration)	6.2 (30.8)	4.9 (22.5)	0.899
Ln(NT-proBNP concentration)	254.7 (190)	57.4 (64.7)	1.554
Ln(CRP concentration)	22 (113)	1.7 (64)	0.339
LDL-C concentration	3.23 ± 1.05	3.57 ± 0.85	−0.351
Ln(triglyceride concentration)	1.36 (48.3)	1.54 (52.8)	−0.262

Effective sample size after weighting: 167.8 cases and 163.8 controls. Mean weight 1; SD 0.269 for cases and 0.305 for controls.

Table S4. Characteristics of cases and controls after covariate balancing undertaken to estimate the association between plasma homocysteine concentrations [1] and NVAF. Data are weighted percentages, or mean ± standard deviation, or geometric mean (%coefficient of variation) for ln-transformed variables. Standardized mean differences (d) < 0.1 indicate irrelevant differences.

	**Cases**	**Controls**	**d**
N	180	179	---
Variables used for balancing			
MTHFR 677C>T (rs1801133)			
CC	44.6	44.6	0.000
CT	43.2	43.2	0.000
TT	12.3	12.3	0.000
KCNE1 A>G (rs1805127)			
AA	22.0	22.0	0.000
AG	47.6	47.6	0.000
GG	30.4	30.4	0.000
PITX2 C>T (2200733)			
CC	63.5	63.5	0.000
CT	32.0	32.0	0.000
TT	4.5	4.5	0.000
Diabetes	17.5	17.5	0.000
Body mass index category			
Overweight	55.2	55.2	0.000
Obese	44.8	44.8	0.000
Ln(folate concentration)	14.8 (33.7)	14.8 (43.2)	0.000
Current smoking	17.6	17.6	0.000
Exposure of primary interest			
Ln(plasma homocysteine)	11.7 (26.0)	9.4 (31.5)	0.758
Covariates that could not be balanced			
Age	64 ± 10	49 ± 13	1.382
Men	66.7	34.3	0.681
Hypertension	92.3	48.2	1.096
Systolic blood pressure	147 ± 19	127 ± 12	1.226
Diastolic blood pressure	88 ± 10	78 ± 8	1.051
Ln(creatinine concentration)	88 (25)	73 (21)	0.861
Ln(urea concentration)	6.2 (30.9)	4.9 (22.5)	0.890
Ln(NT-proBNP concentration)	256 (192)	57.5 (64.7)	1.557
Ln(CRP concentration)	2.2 (112)	1.7 (64)	0.337
LDL-C concentration	3.23 ± 1.05	3.57 ± 0.85	−0.354
Ln(triglyceride concentration)	1.36 (48.4)	1.54 (53.3)	−0.263

Effective sample size after weighting: 166.8 cases and 163.5 controls. Mean weight 1; SD 0.281 for cases and 0.308 for controls.

Table S5. Characteristics of cases and controls after covariate balancing undertaken to estimate the association between *PITX2 C>T* (rs2200733) [1] polymorphism and NVAF. Data are weighted percentages, or mean ± standard deviation, or geometric mean (% coefficient of variation) for ln-transformed variables. Standardized mean differences (d) < 0.1 indicate irrelevant differences.

	**Cases**	**Controls**	**d**
N	180	179	---
Variables used for balancing			
KCNE1 A>G (rs1805127)			
AA	22.0	22.0	0.000

AG	47.6	47.6	0.000
GG	33.4	33.4	0.000
MTHFR 677C>T (rs1801133)			
CC	44.6	44.6	0.000
CT	43.2	43.2	0.000
TT	12.3	12.3	0.000
Diabetes	17.5	17.5	0.000
Body mass index category			
Overweight	55.2	55.2	0.000
Obese	44.8	44.8	0.000
Ln(folate concentration)	14.8 (33.6)	14.8 (43.3)	0.000
Current smoking	17.6	17.6	0.000
Exposure of primary interest			
PITX2 C>T (rs2200733) variant carrier	42.7	27.7	0.316
PITX2 C>T (rs2200733) wild type	57.3	72.3	−0.316
Covariates that could not be balanced			
Age	64 ± 10	49 ± 13	1.359
Men	66.6	35.0	0.665
Hypertension	91.8	47.6	1.097
Systolic blood pressure	147 ± 19	127 ± 12	1.237
Diastolic blood pressure	88 ± 10	78 ± 8	1.056
Ln(homocysteine concentration)	11.7 (26.0)	9.4 (31.3)	0.745
Ln(creatinine concentration)	88 (24)	73 (21)	0.851
Ln(urea concentration)	6.2 (30.4)	4.9 (22.2)	0.877
Ln(NT-proBNP concentration)	251 (190)	57 (66)	1.536
Ln(CRP concentration)	2.2 (109)	1.7 (65)	0.337
LDL-C concentration	3.24 ± 1.05	3.58 ± 0.86	−0.365
Ln(triglyceride concentration)	1.36 (48.5)	1.54 (53.5)	−0.265

Effective sample size after weighting: 169.5 cases and 169.8 controls. Mean weight 1; SD 0.249 for cases and 0.233 for controls.

Table S6. Characteristics of cases and controls after covariate balancing undertaken to estimate the association between *KCNE1 A>G* (rs1805127) [1] polymorphism and NVAF. Data are weighted percentages, or mean ± standard deviation, or geometric mean (% coefficient of variation) for ln-transformed variables. Standardized mean differences (d) < 0.1 indicate irrelevant differences.

	**Cases**	**Controls**	**d**
N	180	179	---
Variables used for balancing			
MTHFR 677C>T (rs1801133)			
CC	44.6	44.6	0.000
CT	43.2	43.2	0.000
TT	12.3	12.3	0.000
PITX2 C>T (2200733)			
CC	63.5	63.5	0.000
CT	32.0	32.0	0.000
TT	4.5	4.5	0.000
Diabetes	17.5	17.5	0.000
Body mass index category			
Overweight	55.2	55.2	0.000
Obese	44.8	44.8	0.000
Ln(folate concentration)	14.8 (33.5)	14.8 (42.9)	0.000
Current smoking	17.6	17.6	0.000

Exposure of primary interest			
KCNE1 A>G (rs1805127) variant carrier	75.5	81.2	−0.139
KCNE1 A>G (rs1805127) wild type	24.5	18.8	0.139
Covariates that could not be balanced			
Age	64 ± 10	49 ± 13	1.375
Men	66.5	34.6	0.671
Hypertension	92.4	48.3	1.095
Systolic blood pressure	147 ± 19	127 ± 12	1.225
Diastolic blood pressure	88 ± 10	78 ± 8	1.058
Ln(homocysteine concentration)	11.7 (26.2)	9.4 (31.5)	0.752
Ln(creatinine concentration)	88 (24)	73 (21)	0.841
Ln(urea concentration)	6.2 (30.6)	4.9 (22.7)	0.877
Ln(NT-proBNP concentration)	256 (190)	57 (65)	1.557
Ln(CRP concentration)	2.2 (112)	1.7 (65.7)	0.317
LDL-C concentration	3.24 ± 1.05	3.57 ± 0.84	−0.345
Ln(triglyceride concentration)	1.36 (48.5)	1.55 (52.7)	−0.268

Effective sample size after weighting: 167.9 cases and 165.3 controls. Mean weight 1; SD 0.268 for cases and 0.287 for controls.

C.2. Principal components analysis to reduce dimensionality of covariates that could not be adequately balanced (Figure S3)

Of the covariates that could not be adequately balanced by optimization-based weighting, we considered that age, sex, tHcy, and CRP should be treated as individual covariates owing to their importance in the current setting. All other covariates were submitted to principal components analysis (PCA): three components were identified (eigenvalues 2.88, 1.52, and 1.12; the fourth dimension had an eigenvalue of 0.65) that cumulatively explained 69.0% of the variance and were termed "Blood pressure" (BP), "Renal-BNP", and "Lipids" (Figure S3).

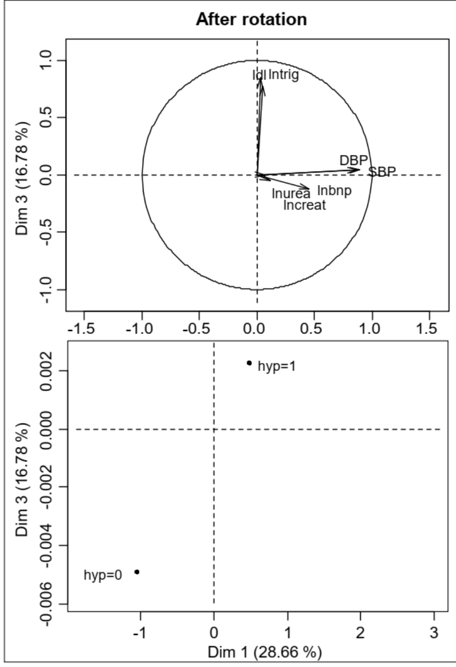

Dimensions	Component	Variance	Proportion
Dimension 1	Blood pressure	2.29	28.7%
Dimension 2	Renal-BNP	1.88	23.5%
Dimension 3	Lipid	1.34	16.8%
Cumulative			69.0%

Variable	BP	Renal-BNP	Lipid
Systolic BP	**0.80**	0.00	0.00
Diastolic BP	**0.77**	0.00	0.00
Ln(BNP)	0.21	**0.32**	0.01
Ln(creatinine)	0.01	**0.67**	0.00
Ln(urea)	0.01	**0.69**	0.00
Ln(triglyceride)	0.00	0.01	**0.71**
LDL-C	0.00	0.05	**0.61**
Hypertension	**0.50**	0.13	0.27

Figure S3. Summary of the principal components analysis: squared loadings for the continuous variables (upper left: urea [lnurea], creatinine [lncreat], systolic and diastolic blood pressure [SBP, DBP],

low-density lipoprotein cholesterol [LDL-C], triglycerides [lntrig], and NT-proBNP [lnbnp]), and the categorical variables (lower left: hypertension [hyp]). Three components were identified that explained 69% of the variance (**upper right**). Factor loadings (**lower right**) indicate components "BP (blood pressure)" (SBP + DBP + hypertension), "renal-BNP" (creatinine + urea + ln[BNP]), and "lipid" (LDL-C and ln[triglycerides]).

C.3 Partial correlation analysis of covariates

To explore the relationship between covariates considered in conventional case–control analysis, we conducted partial correlation analysis (Table S7): tHcy positively correlated with age, male sex, and Renal-BNP (higher values are worse). Older age was associated with female sex and higher values of Renal-BNP and BP (higher values are worse). Male sex was associated with higher values of Renal-BNP and of BP, whereas Renal-BNP inversely correlated with BP (partial correlations were estimated with additional control for folate concentrations).

Table S7. Summary of the partial correlation analysis of covariates (additional control for folate concentrations). Data are correlation coefficients; respective P-values.

	Ln(tHcy)	Age	Sex	Renal-BNP	BP	Lipid	Ln(CRP)
Ln(tHcy)	.	0.280; <0.001	0.139; 0.009	0.147; 0.005	0.039; 0.475	0.036; 0.503	0.075; 0.163
Age	0.280	.	−0.253; <0.001	0.499; 0.001	0.542; 0.001	−0.077; 0.146	−0.028; 0.604
Sex	0.139	−0.253	.	0.446; 0.001	0.217; 0.001	0.101; 0.058	0.009; 0.866
Renal-BNP	0.147	0.499	0.446	.	−0.378; <0.001	−0.005; 0.921	0.116; 0.029
BP	0.039	0.542	0.2167	−0.378	.	0.030; 0.575	0.093; 0.082
Lipid	0.036	−0.078	0.1011	−0.005	0.030	.	−0.065; 0.221
Ln(CRP)	0.075	−0.028	0.0090	0.1160	0.093	−0.065	.

C.4. Bias-corrected estimates

With correction for a hypothetical residual confounding effect (RR = 1.50 or 0.67, with 3:1 imbalance in cases vs. controls [0.60 vs. 0.20]) and misclassification of the outcome (NVAF) (sensitivity of classification is 77%), the adjusted estimates were moderately altered, but qualitative conclusions were not changed: the *PITX2 C>T* variant allele remained strongly associated with the outcome; other SNPs and tHcy remained unassociated with the outcome (Table S8).

Table S8. Fully adjusted estimates (Table 2 in the main text) in the form of RR were corrected for misclassification bias (outcome), and residual confounding (RC). Bias-corrected estimates are shown with the lower (in the case of RR > 1.0) or upper (in the case of RR < 1.0) 95% confidence limit (CL).

	**Fully Adjusted**	**Bias Corrected (RC RR = 1.5)**	**Bias Corrected (RC RR = 0.67)**
	**RR (95%CI)**	**RR (lower/upper 95% CL)**	**RR (lower/upper 95% CL)**
MTHFR677 variant vs. wild type	0.99 (0.70–1.40)	0.85 (1.18)	1.17 (0.86)
Homocysteine (33% higher)	1.00 (0.83–1.20)	0.85 (1.18)	1.16 (0.86)
PITX2 variant vs. wild type	1.55 (1.06–2.25)	1.32 (1.18)	1.81 (1.16)
KCNE1 variant vs. wild type	0.88 (0.61–1.26)	0.72 (1.18)	0.99 (0.86)

Supplementary Material D. Additional Results III: Principal Components Analysis (PCA) for the Purpose of Mediation and MR/IV Analysis

We deemed that, in the mediation and one-sample MR/IV analysis, age, sex, folate concentrations, and *KCNE1 112A>G* and *PITX2 C>T* SNPs have to be treated as individual variables owing to their conceptual importance, while others (actual blood pressure, pre-existing hypertension, NT-ProBNP, creatinine, urea, CRP, triglycerides, LDL-C, diabetes, fasting glucose, BMI category, and current smoking) were submitted to a PCA. Five components (dimensions) were identified with eigenvalues > 1.0 (3.14, 1.61, 1.47, 1.25, and 1.15, respectively; the sixth dimension had an eigenvalue of 0.96) and were named: "Blood pressure" (BP) (actual systolic and diastolic BP + pre-existing hypertension), "Lipid" (LDL-C + triglycerides), "Diabetes" (fasting glucose + pre-existing diabetes), "BMI-CRP" (body mass index category + CRP + current smoking), and "Renal-BNP" (creatinine + urea + NT-proBNP). For the component "Lipid", lower values are "worse", whereas for all other components, higher values are "worse" (i.e., associated with NVAF). Cumulatively, these dimensions/components explained 65.4% of the variance (Figure S9).

In partial correlation analysis (additional control for folate concentrations), tHcy positively correlated with age, male sex, and "Renal-BNP" (higher values are worse). Other correlations are depicted in Table S9. Of note, "Renal-BNP" was higher (worse) in men than in women and inversely correlated with "Blood pressure" and "Diabetes", but note that these are partial correlations; in simple bivariate correlations, none of the components identified in PCA mutually correlated (as expected with a successful PCA).

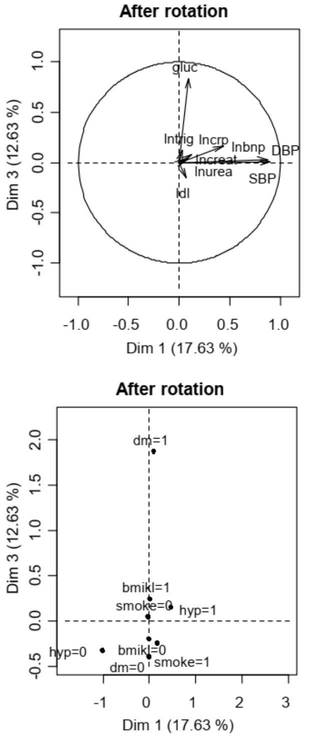

Dimensions	Component	Variance	Proportion
Dimension 1	Blood pressure	2.29	17.6%
Dimension 2	Lipid	1.38	10.6%
Dimension 3	Diabetes	1.64	12.6%
Dimension 4	BMI-CRP	1.32	10.2%
Dimension 5	Renal-BNP	1.86	14.3%
Cumulative			65.4%

Variable	BP	Lipid	DM	BMI-CRP	Renal-BNP
Systolic BP	**0.81**	0.00	0.00	0.00	0.00
Diastolic BP	**0.77**	0.00	0.00	0.00	0.00
Ln(BNP)	0.19	0.01	0.03	0.04	**0.29**
Ln(creatinine)	0.01	0.00	0.00	0.00	**0.67**
Ln(urea)	0.02	0.00	0.01	0.00	**0.69**
Ln(triglycerides)	0.00	**0.68**	0.02	0.00	0.00
LDL-C	0.00	**0.57**	0.02	0.00	0.03
Fasting glucose	0.01	0.00	**0.71**	0.00	0.01
Ln(CRP)	0.00	0.00	0.00	**0.47**	0.05
Hypertension	**0.47**	0.00	0.05	0.00	0.11
Diabetes	0.00	0.00	**0.75**	0.00	0.00
Current smoker	0.01	0.02	0.01	**0.50**	0.01
BMI category	0.00	0.09	0.05	**0.31**	0.00

Figure S4. Summary of the principal components analysis: squared loadings for the continuous variables (upper left: urea [lnurea], creatinine [lncreat], systolic and diastolic blood pressure [SBP, DBP], low-density lipoprotein cholesterol [LDL-C], triglycerides [lntrig], fasting glucose (gluc) and NT-

proBNP [lnbnp], and CRP [lncrp]) and the categorical variables (lower left: hypertension [hyp], diabetes [dm], current smoking [smoke], and body mass index class [bmikl]), variance explained (upper right), and factor loadings (lower right).

Table S9. Summary of the partial correlation analysis (additional control for folate concentrations). Data are correlation coefficients; respective *p*-values.

	Ln(Hcy)	Age	Sex	Renal-BNP	BP	Lipid	Diabetes	BMI-CRP
Ln(Hcy)	.	0.270; <0.001	0.141; 0.008	0.154; 0.004	0.046; 0.385	0.017; 0.756	0.051; 0.338	0.077; 0.151
Age	0.270	.	−0.256; <0.001	0.499; <0.001	0.540; <0.001	−0.063; 0.235	0.313; <0001	−0.035; 0.518
Sex	0.141	−0.256	.	0.442; <0.001	0.224; <0.001	0.095; 0.076	0.121; 0.023	0.051; 0.345
Renal-BNP	0.154	0.499	0.442	.	−0.368; <0.001	−0.011; 0.838	−0.219; <0.001	−0.024; 0.648
BP	0.046	0.540	0.224	−0.368	.	0.020; 0.706	−0.205; <0.001	−0.000; 0.999
Lipid	0.017	−0.063	0.095	−0.011	0.020	.	0.012; 0.822	−0.008; 0.885
Diabetes	0.051	0.313	0.121	−0.219	−0.205	0.012	.	−0.001; 0.990
	0.077	−0.035	0.051	−0.024	−0.000	−0.008	−0.001	.

Supplementary Material E. Additional Results IV: Outline of the Mediation Analysis

To assess (forward) the association between the *MTHFR 677C>T* (exposure)/tHcy (exposure or mediator) and NVAF in mediation analysis, we considered that the following individual variables and latent variables identified in PCA should be treated as potential confounders of the exposure–mediator, mediator–outcome, and exposure–outcome relationship: (i) age, sex, "Diabetes" (pre-existing diabetes + fasting glucose), "BMI-CRP" (body mass index category [obese or overweight] + CRP + current smoking), and "Lipid" (triglycerides + LDL-C), since these are (or subsume) classical conventional risk factors for atrial fibrillation (AF) [1–6], while there has been no GWAS association between *MTHFR 677C>T* polymorphism and these conditions [7]. By this virtue, they are unlikely mediators or colliders on the assessed paths (*MTHFR*/tHcy-NVAF; (ii) "Blood pressure" (pre-existing hypertension + actual blood pressure). Hypertension is a known risk factor for AF [1–6]. The *MTHFR* SNP has been reported GWAS-associated with hypertension, but this has been challenged [7], and quality conventional and MR analyses have rejected the causal role of tHcy [8,9] in arterial hypertension; (iii) *PITX2 C>T* and *KCNE1 112A>G* SNPs. As elaborated in the Introduction, the former is a well-established causal factor in AF, while contradictory results have been reported for the latter; and (iv) plasma folate concentrations, for their known effect on tHcy levels.

We considered that the role of the variable "Renal-BNP" (NT-proBNP + urea + creatinine) was not straightforwardly clear: NT-proBNP and renal function (indicated by urea and creatinine, while chronic kidney disease was an exclusion criterion) are known risk factors for AF [1–6], hence "Renal-BNP" could be a confounder. On the other hand, there is quality MR and conventional epidemiological evidence of the causal effect of tHcy on NT-proBNP levels, as well as on (the deterioration of) renal function [10–12]; hence, "Renal-BNP" could be a mediator. Finally, it is plausible that AF could reflect on NT-proBNP levels and/or on the renal function and, consequently, on the variable "Renal-BNP" that subsumes these elements; hence, "Renal-BNP" could also be a collider (e.g., affected both by tHcy and NVAF). Confusing mediators, colliders, and confounders may result in seriously biased estimates. To clarify the likely most appropriate role of "Renal-BNP", we

conducted one-sample MR/IV analysis with *PITX2 C>T* polymorphism as the instrument, atrial fibrillation (NVAF) as the exposure, and "Renal-BNP" and each of the variables that it subsumed (ln[NT-proBNP], ln[creatinine], and ln[urea]) as the outcomes. Following general recommendations for one-sample MR/IV analysis [13], we (i) included age, sex, *KCNE1 112A>G* polymorphism, ln(tHcy concentration), and "Lipid" as covariates, i.e., possible confounders of the instrument–exposure and instrument–outcome relationships; (ii) avoided the inclusion of variables that could be on a path between the exposure and the outcome (mediators, colliders), i.e., latent variables "Blood pressure", "Diabetes", and "BMI-CRP". The analysis was based on two-stage predictor substitution estimators, as implemented in package OneSampleMR [14]. The method first regresses exposure (NVAF) on the instrument and covariates (confounders), which generates predicted values of the exposure. The second stage model is then fitted, in which the outcome is regressed on the predicted values of the exposure (and covariates). The procedure estimates mean differences as measures of association (effect) between the exposure (or the covariates) and the outcome. For ln-transformed outcomes, the association is quantified as a geometric means ratio (GMR). As summarized in Table S10, the (i) exposure—NVAF—did not appear associated with any outcome; (ii) tHcy was associated with higher "Renal-BNP" and higher ln(creatinine) and numerically tended to be associated with higher ln(urea); (ii) older age and male sex were associated with higher "Renal-BNP" and higher ln(creatinine) and ln(urea) (age was also associated with higher NT-proBNP).

Table S10. Summary of one-sample MR/IV analysis of outcomes "Renal-BNP" and variables subsumed in this latent variable (NT-proBNP, creatinine, and urea concentrations), with *PITX2 C>T* polymorphism as the instrument and non-valvular atrial fibrillation (NVAF) as the exposure.

	Outcome Is "Renal-BNP"		Outcome Is Ln(NT-proBNP)	
	Mean Δ (95%CI)	P	GMR (95%CI)	P
Effect of exposure				
NVAF (vs. no)	−0.52 (−2.16, 1.15)	0.532	1.59 (0.43–5.57)	0.504
Covariate effects				
Age	0.05 (0.01, 0.08)	0.008	1.05 (1.02–1.07)	<0.001
Male sex	1.11 (0.55, 1.67)	<0.001	1.07 (0.73–1.57)	0.739
Ln(tHcy)	0.52 (0.07, 0.97)	0.024	1.23 (0.90–1.69)	0.193
KCNE1 variant allele	0.15 (−0.12, 0.42)	0.278	0.93 (0.74–1.16)	0.513
"Lipid"	−0.05 (−0.23, 0.13)	0.580	0.94 (0.83–1.06)	0.313
	Outcome is Ln(creatinine)		Outcome is Ln(urea)	
	GMR (95%CI)	P	GMR (95%CI)	P
Effect of exposure				
NVAF (vs. no)	0.59 (0.11–3.05)	0.532	1.01 (0.71–1.44)	0.938
Covariate effects				
Age	1.05 (1.01–1.09)	0.009	1.01 (1.00–1.02)	0.031
Male sex	3.03 (1.73–5.32)	<0.001	1.14 (1.02–1.28)	0.017
Ln(tHcy)	1.68 (1.07–2.64)	0.024	1.08 (0.99–1.18)	0.071
KCNE1 variant allele	1.16 (0.89–1.52)	0.272	1.02 (0.96–1.09)	0.511
"Lipid"	0.95 (0.79–1.14)	0.580	0.99 (0.96–1.02)	0.538

Based on the elaborated literature data and the analysis in Table S10, we considered that "Renal-BNP" is unlikely a collider on a path between *MTHFR 677C>T* polymorphism, tHcy, and NVAF, whereas on a path between *PTIX2 C>T* (exposure) and tHcy as the outcome, with NVAF as a mediator, it could be a collider. Consequently, we conceived several mediation models to be evaluated (Figure S5): (i) a causal mediation model to assess

the association between the *MTHFR 677C>T* variant allele as exposure and NVAF (outcome), with tHcy as a mediator (Figure S5A). The model is fitted twice, once with "Renal-BNP" as a confounder and once without it in the model, since it is possibly a mediator; (ii) a traditional mediation model to assess the association between the *MTHFR 677C>T* variant allele (exposure) and NVAF (outcome), with two consecutive mediators, tHcy and "Renal-BNP" (Figure S5B); (iii) a causal mediation model with tHcy exposure, "Renal-BNP" as a mediator, and NVAF outcome (Figure S5C). The model is fitted in all subjects and separately in *MTHFR* wild-type subjects and in *MTHFR* variant carriers; and (iv) a causal mediation model to assess reverse tHcy-NVAF association: the *PITX2 C>T* variant allele is the exposure, NVAF is the mediator, and tHcy is the outcome (Figure S5D). The model is fitted twice, with "Renal-BNP" as a confounder and without it, since it is likely a collider.

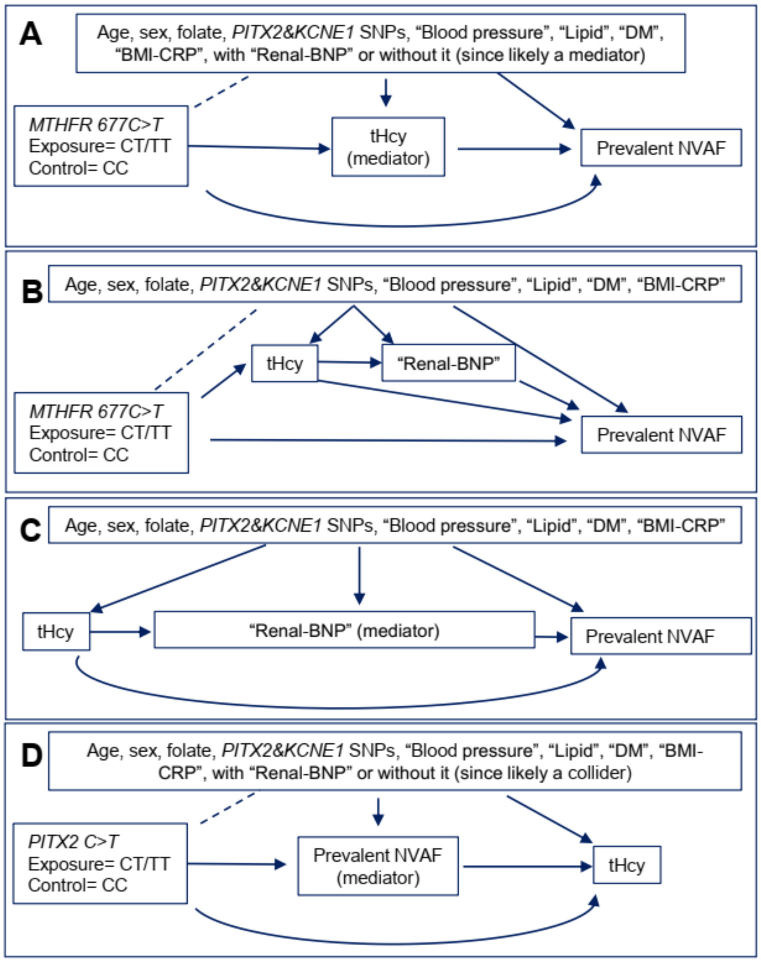

Figure S5. Mediation models for estimation of forward (**A**–**C**) and reverse (**D**) association between plasma homocysteine (tHcy) and non-valvular atrial fibrillation (NVAF) outlined as simplified directed acyclic graphs. Variables denoted in quotation marks are latent variables from the principal components analysis (Supplementary D).

References

1. Hindricks G, Potpara T, Dagres N, et al. 2020 ESC Guidelines for the diagnosis and management of atrial fibrillation developed in collaboration with European association of cardio-thoracic surgery (EACTS). Eur Heart J 2021, 42, 373-498.

2. Wang N, Yu Y, Sun Y, et al. Acquired risk factor and incident atrial fibrillation according to age and genetic predisposition. Eur Heart J 2023, 44, 4982-4993.
3. Wang J, Jiang C, Li S, et al. Systolic blood pressure time in target range and incident atrial fibrillation in patients with hypertension: insight from the SPRINT trial. Hypertension 2023, 80, 2306-2314.
4. Young LJ, Antwi-Boasiako S, Ferrall J. Wold LE, Mohler PJ, El Refaey M. Genetic and non-genetic risk factors associated with atrial fibrillation. Life Sci 2022, 299, 120529. https://doi.org.10.1016/j.lfs.2022.120529
5. Elliott AD, Middeldorp ME, Van Gelder IC, Albert CM, Sanders P. Author Correction: Epidemiology and modifiable risk factors for atrial fibrillation. Nat Rev Cardiol 2023, 20, 429.
6. Schnabel RB, Larson MG, Yamamoto JF, et al. Relations of biomarkers of distinct pathophysiological pathways and atrial fibrillation incidence in the community. Circulation 2010, 121, 200-207.
7. GWAS Catalog. The NHGRI-EBI Catalog of human genome-wide association studies. Available at: GWAS Catalog, Accessed May 21, 2025.
8. Meng H, Huang S, Yang Y, He, Fei L, Xing Y. Association between MTHFR polymorphisms and the risk of essential hypertension: an updated meta-analysis. Front Genet 2021, 12, 698590. doi: 10.3389/fgene.2021.698590
9. Borges MC, Hartwig FP, Oliveira IO, Horta BL. Is there a causal role of homocysteine in blood pressure? A mendelian randomization study. Am J Clin Nutrition 2016, 103, 39-49.
10. Park SP, Lee S, Kim Y, et al. Causal effects of homocysteine, folate and cobalamin on kidney function: a Mendelian randomization study. Nutrients 2021, 13, 906. https://doi.org/10.3390/nu13030906
11. Gueant Rodriguez RM, Spada R, Pooya S, et al. Homocysteine predict increase NT-proBNP through impaired fatty acid oxidation. Int J Cardiol 2013, 167, 768-775.
12. Tan X, Tang F, Tian W, et al. Homocysteine metabolism, subclinical myocardial injury, and cardiovascular mortality in the general population. J Am Coll Cardiol: Asia 2024; 4:609-620.
13. Burgess S, Davey Smith G, Davies NM, et al. Guidelines for performing Mendelian randomization investigations: update for summer 2023, Wellcome Open Res 2023, 4, 186. https://doi.org/10.12688/wellcomeopenres.15555.3
14. Palmer T, Spiller W, Sanderson E. OneSampleMR: One sample Mendelian randomization and instrumental variable analyses. MRC Integrative Epidemiology Unit, University of Bristol, Bristol UK, 2023. https://remlapmot.github.io/OneSampleMR/.

Supplementary Material F. Additional Results V: Choice of Covariates (Potential Confounders) in the MR/IV Analysis

The choice of covariates (potential confounders of the instrument–exposure and instrument–outcome associations) in the MR/IV analysis of the forward association between tHcy and NVAF (*MTHFR 677C>T* SNP dichotomized as variant carriage vs. wild type as the instrument) and the reverse association between tHcy and NVAF (*PITX2 C>T* SNP dichotomized as variant carriage vs. wild type as the instrument; NVAF is the exposure; and Ln[tHcy] is the outcome) was based on the following rationale, in line with general recommendations for one-sample MR/IV analysis [1]:

i. ″Renal-BNP″ from PCA should not be included in either model, since it is a likely mediator of the effect of tHcy on NVAF and a possible collider for the reverse association (may be affected by the outcome [tHcy]);
ii. Candidates for inclusion are patient characteristics imbalanced between *MTHFR* or *PITX2* variant carriers and wild-type subjects (standardized mean difference ≥ 0.1), unless there is evidence or biological rationale that would render them likely mediators of colliders on the path between the exposures and the outcomes.

MTHFR 677C>T variant carriers and wild-type subjects appeared imbalanced regarding the prevalence of *PITX2 C>T* variant carriers (d = −0.155) and "Lipid" (d = 0.134) (Table S11). Since low triglycerides and LDL-C are classical risk factors for AF [2] and are unlikely to be affected by tHcy, these two variables were included in the model for forward tHcy-NVAF association. Also included was "sex", although the imbalance did not reach d = 0.100 (d = 0.093), but "sex" has a strong effect on the risk of atrial fibrillation [1].

Table S11. Characteristics of *MTHFR 677C>T* variant carriers and wild-type subjects. Data are mean±SD and median (quartiles) or counts (%). Shown are standardized mean differences (d) for variant carriers vs. wild-type subjects. Values < 0.1 indicate irrelevant differences.

	Variant Carrier	Wild Type	d
N	199	160	
Potential confounders			
Age	56 ± 13; 60 (45–67)	55 ± 15; 59 (45–67)	0.067
Male sex	105 (52.8)	77 (48.1)	0.093
Ln(folate)	2.69 ± 0.37; 2.65 (2.42, 2.95)	2.72 ± 0.39; 2.72 (2.42, 2.95)	−0.079
PITX2 C>T variant	66 (33.2)	65 (40.6)	−0.155
KCNE1 112A>G variant	154 (77.4)	126 (78.7)	−0.033
"Blood pressure"	0.04 ± 1.51; −0.02 (−1.03, 1.09)	−0.05 ± 1.53; −0.08 (−1.25, 1.20)	0.057
"Diabetes"	−0.01 ± 1.31; −0.38 (−0.76, 0.07)	0.01 ± 1.26; −0.43 (−0.75, 0.11)	−0.009
"BMI-CRP"	−0.03 ± 1.15; −0.23 (−0.86, 0.53)	0.04 ± 1.15; −0.14 (−0.77, 0.46)	−0.056
"Lipid"	0.07 ± 1.21; 0.07 (−0.80, 0.89)	−0.09 ± 1.14; −0.02 (−0.94, 0.68)	0.134

PITX2 C>T variant carriers and wild-type subjects appeared imbalanced regarding the prevalence of *MTHFR 677C>T* variant carriers (d = −0.160), *KCNE1 112A>G* variant carriers (d = 0.203), "Blood pressure" (d = 0.196) from PCA (actual blood pressure + preexisting hypertension), and "BMI-CRP" (d = 0.163) from PCA (BMI category + current smoking + CRP) (Table S12). These variables were included in the model for reverse tHcy-NVAF association: (i) the *MTHFR* SNP affects tHcy (exposure); (ii) impact of the *KCNE1* is uncertain; iii) actual blood pressure/hypertension (as "Blood pressure") are known risk factors for AF [2] and are most likely not affected by tHcy [3,4]; and (iii) obesity, smoking, and CRP (as "BMI-CRP") are all known risk factors for AF [1], and there has been no indication thus far that they are affected by tHcy [5].

Table S12. Characteristics of *PITX2 C>T* variant carriers and wild-type subjects. Data are mean±SD and median (quartiles) or counts (%). Shown are standardized mean differences (d) for variant carriers vs. wild-type subjects. Values < 0.1 indicate irrelevant differences.

	**Variant Carrier**	**Wild Type**	**d**
N	131	228	
Potential confounders			
Age	56 ± 13; 60 (47-67)	56 ± 14; 59 (44, 66)	0.052
Male sex	67 (51.1)	115 (50.4)	0.014
Ln(folate)	2.70 ± 0.36; 2.67 (2.44, 2.95)	2.69 ± 0.39; 2.68 (2.41, 2.95)	0.029
MTHFR 677 C>T variant	66 (50.4)	133 (58.3)	−0.160

KCNE1 112A>G variant	109 (83.2)	171 (75.0)	0.203
"Blood pressure"	0.19 ± 1.46; 0.21 (−0.94, 1.30)	−0.11 ± 1.54; −0.06 (−1.25, 1.02)	0.196
"Diabetes"	0.03 ± 1.30; −0.41 (−0.75,−0.30)	−0.02 ± 1.28; −0.40 (−0.76, 0.03)	0.035
"BMI-CRP"	0.12 ± 1.19; −0.06 (−0.81, 0.65)	−0.07 ± 1.12; −0.23 (−0.83, 0.43)	0.163
"Lipid"	−0.04 ± 1.16; 0.06 (−0.88, 0.84)	0.02 ± 1.19; −0.05 (−0.80, 0.75)	−0.055

References

1. Burgess S, Davey Smith G, Davies NM, et al. Guidelines for performing Mendelian randomization investigations: update for summer 2023, Wellcome Open Res 2023, 4, 186. https://doi.org/10.12688/wellcomeopenres.15555.3
2. Hindricks G, Potpara T, Dagres N, et al. 2020 ESC Guidelines for the diagnosis and management of atrial fibrillation developed in collaboration with European association of cardio-thoracic surgery (EACTS). Eur Heart J 2021, 42, 373-498.
3. Meng H, Huang S, Yang Y, He, Fei L, Xing Y. Association between MTHFR polymorphisms and the risk of essential hypertension: an updated meta-analysis. Front Genet 2021, 12, 698590. doi: 10.3389/fgene.2021.698590
4. Borges MC, Hartwig FP, Oliveira IO, Horta BL. Is there a causal role of ho-mocysteine in blood pressure? A mendelian randomization study. Am J Clin Nutrition 2016, 103, 39-49.
5. GWAS Catalog. The NHGRI-EBI Catalog of human genome-wide associa-tion studies. Available at: GWAS Catalog, Accessed May 21, 2025.

Supplementary Material G. A Hypothesis: In *MTHFR 677C>T* Variant Carriers, tHcy might Contribute to Both Higher and Lower Probability of NVAF

The present hypothesis is based on the existing data outlined in Figure S6—a cohort study in Sweden by Calderon-Larranaga et al. [1] (Figure S6A) and current observations from mediation analyses depicted in Table 5, Model 3 (Figure S6B).

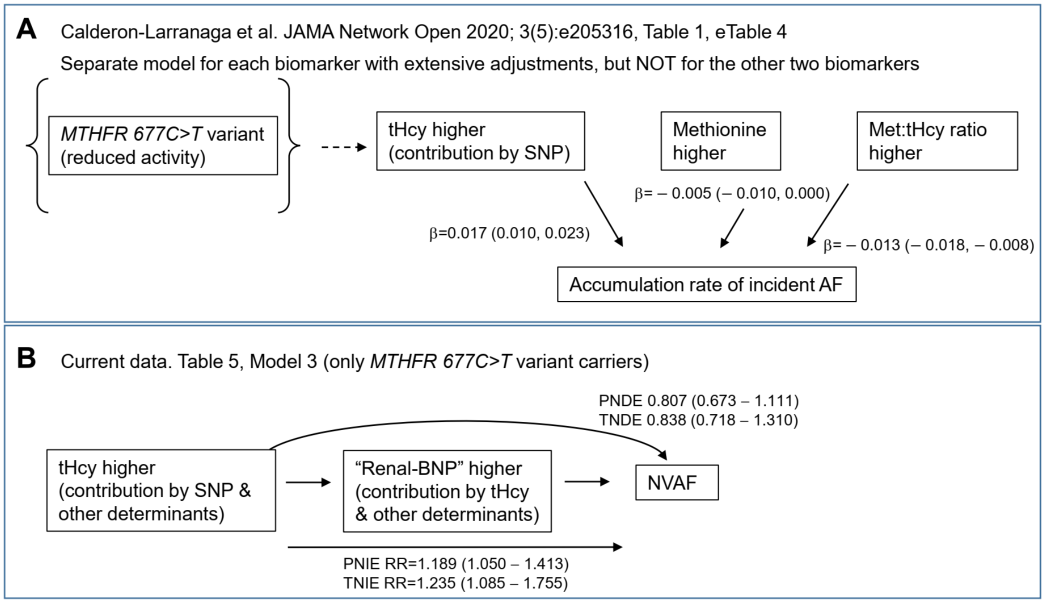

Figure S6. **(A)** Summary of a published study [1] indicating that higher tHcy increases the risk of incident atrial fibrillation [NV(AF)], that higher Methionine (Met) tends to reduce it, and that higher

Met:tHcy ratio reduces it. (**B**) Summary of a current analysis in *MTHFR* variant carriers indicating that (higher) tHcy associates with higher probability of NVAF "through" its effect on "Renal-BNP" (pure and total natural indirect effects, PNIE and TNIE), but also indicating the possibility of an inverse tHcy-NVAF association (through some other path) (pure and total natural direct effects, PNDE and TNDE).

The proposed concept is outlined in Figure S7. 1. Present data indicate that in adult *MTHFR 677C>T* variant carriers, the variant allele contributes (along with other factors) to higher values of tHcy. While the impact of "other factors" may vary over time (including variations in methionine dietary intake), the genetic contribution is a long-term, mild–moderately increased tHcy. 2. Present data depict a link between tHcy and a higher probability of NVAF mediated "through" the effect of tHcy on the "Renal-BNP" component from PCA. 3. The published study [1] indicated that higher methionine (due to, e.g., dietary intake and/or other factors that may vary over time) associates with a lower probability of incident AF. 4. The first "hypothesized" part is that long-term, genetically mild increased tHcy contributes to higher methionine. Assuming that dietary methionine intake in the earlier periods of life is unlikely to materially contribute to a "higher level" of methionine, consistently genetically driven higher tHcy may eventually contribute to higher methionine in these periods of life despite the reduced activity of MTHFR, e.g., through the betaine-homocysteine methyltransferase in the liver and kidney [2], since the substrate (tHcy) is continuously available at some excess level. 5. The second "hypothesized" part includes two elements: (i) in this context, the Met:tHcy ratio is a consequent "by-stander", i.e., the reported [1] association between the ratio and reduced risk of AF is actually only a reflection of the "effect" of higher methionine; (ii) consequent to its contribution to higher methionine, at a given value of the Met:Hcy ratio, higher tHcy actually associates with higher methionine levels.

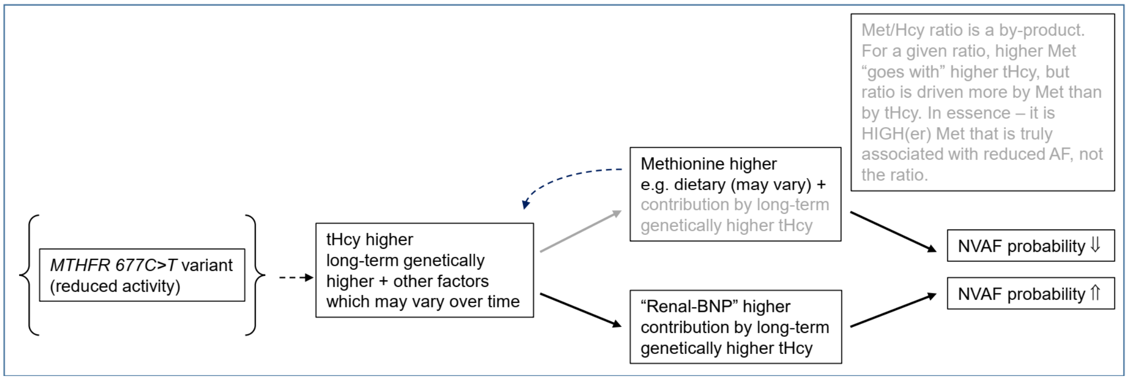

Figure S7. The hypothesized concept of a possible dual effect of tHcy (and, implicitly so, of the *MTHFR 677C>T* polymorphism) on the probability of atrial fibrillation (NVAF) to increase it (e.g., through "Renal-BNP"), as observed in the mediation analysis in the present study, or to decrease it through a hypothesized contribution to higher methionine levels, which have been reported [1] to be associated with a reduced risk of AF. Black font and arrows depict the observed data; gray font and arrows depict hypothesized elements of the concept. See text for explanation.

By this virtue, (higher) tHcy (driven genetically and by other factors) might affect the probability of NVAF in two opposite directions: (i) to increase it (e.g., "through" "Renal-BNP"), which might be a "stronger" effect, as indicated by the values of PNIE and TNIE in the current mediation analysis depicted in Figure S6B; (ii) to decrease it, e.g., "through" the effect on the methionine levels, which might be a "weaker" effect, as indicated by the

values of PNDE and TNDE in Figure S6B. Implicitly, this means also that the link between the *MTHFR 677C>T* variant allele and NVAF might be dual.

The key point of the hypothesis is the assumption that (higher) tHcy contributes to higher methionine, and that, consequently, at each level of the Met:tHcy ratio, higher tHcy is associated with higher methionine levels. We did not measure methionine and cannot directly check this assumption. Calderon-Larranaga et al. [1] did not address the relationship between tHcy, methionine, and their ratio. Also, the estimates of the association of each marker with incident AF were generated without adjustment for the other two, hence it is difficult to deduce whether it is "the ratio" or "methionine" that is actually associated with the reduced risk of AF [1]. However, in Table 1 [1], authors provide 39 triplets of data: median tHcy and methionine and Met:tHcy ratio values across 39 different patient subsets, e.g., by age, by sex, by comorbidity, etc. We used this data in a partial correlation analysis that indicated that, while controlling for the ratio, a higher tHcy associated with higher methionine concentrations (Figure S8A), whereas at both "low" (values 1.5, 1.6 and 1.7) and "high" (values 1.8, 1.9, 2.0) ratios, predicted values of methionine numerically tend to increase with increasing tHcy (Figure S8B). This indicates that, indeed, for a given Met:tHcy ratio, higher tHcy might associate with higher methionine, or, in other words, that it might have the "other path" to NVAF "through" its effect on methionine, which tends to reduce the probability of NVAF.

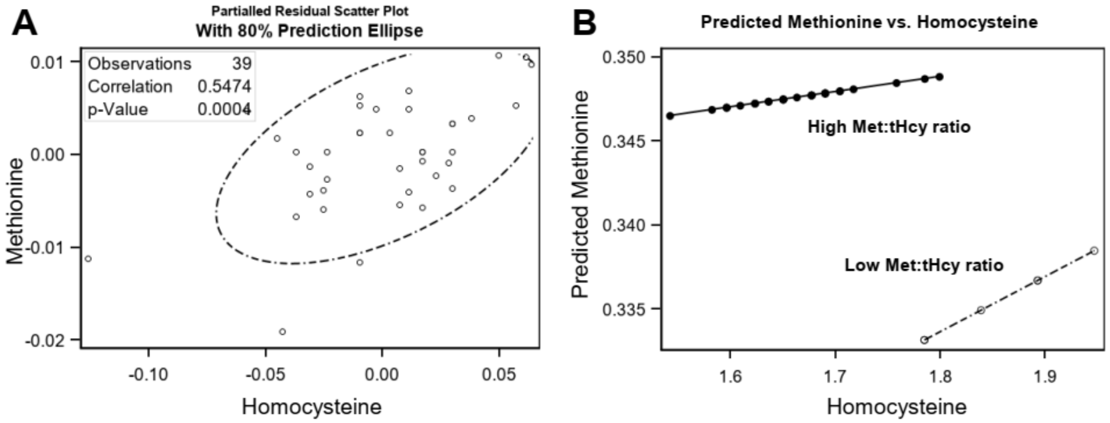

Figure S8. (**A**) Partial correlation analysis: methionine vs. homocysteine controlling for the Met:tHcy ratio. (**B**) Predicted methionine values from a linear model fitted to methionine values, with tHcy (centered), ratio category (low or high), and their interaction.

References

1. Calderon-Larranaga A, Saadeh M, Hooshmand B, et al. (2020) Association of homocysteine, methionine and *MTHFR 677C>T* polymorphism with rate of cardiovascular multimorbidity development in older adults in Sweden. JAMA Network Open 2020, 3, e205316. doi:10.1001/jamanetworkopen.2020.5316
2. McCaddon A, Miller JW. Homocysteine – a retrospective and prospective appraisal. Front Nutr 2023, 10:1179807. doi: 10.3389/fnut.2023.1179807
